# Phylogeny and Origins of Hantaviruses Harbored by Bats, Insectivores, and Rodents

**DOI:** 10.1371/journal.ppat.1003159

**Published:** 2013-02-07

**Authors:** Wen-Ping Guo, Xian-Dan Lin, Wen Wang, Jun-Hua Tian, Mei-Li Cong, Hai-Lin Zhang, Miao-Ruo Wang, Run-Hong Zhou, Jian-Bo Wang, Ming-Hui Li, Jianguo Xu, Edward C. Holmes, Yong-Zhen Zhang

**Affiliations:** 1 State Key Laboratory for Infectious Disease Prevention and Control, Department of Zoonoses, National Institute for Communicable Disease Control and Prevention, Chinese Center for Disease Control and Prevention, Changping, Beijing, China; 2 Wenzhou Center for Disease Control and Prevention, Wenzhou, Zhejiang Province, China; 3 Wuhan Center for Disease Control and Prevention, Wuhan, Hubei Province, China; 4 Yunnan Institute of Endemic Diseases Control and Prevention, Dali, China; 5 Longquan Center for Disease Control and Prevention, Longquan, Zhejiang Province, China; 6 Sydney Emerging Infections and Biosecurity Institute, School of Biological Sciences and Sydney Medical School, The University of Sydney, Sydney, Australia; 7 Fogarty International Center, National Institutes of Health, Bethesda, Maryland, United States of America; US Army Medical Research Institute of Infectious Disease, United States of America

## Abstract

Hantaviruses are among the most important zoonotic pathogens of humans and the subject of heightened global attention. Despite the importance of hantaviruses for public health, there is no consensus on their evolutionary history and especially the frequency of virus-host co-divergence versus cross-species virus transmission. Documenting the extent of hantavirus biodiversity, and particularly their range of mammalian hosts, is critical to resolving this issue. Here, we describe four novel hantaviruses (Huangpi virus, Lianghe virus, Longquan virus, and Yakeshi virus) sampled from bats and shrews in China, and which are distinct from other known hantaviruses. Huangpi virus was found in *Pipistrellus abramus*, Lianghe virus in *Anourosorex squamipes*, Longquan virus in *Rhinolophus affinis*, *Rhinolophus sinicus*, and *Rhinolophus monoceros,* and Yakeshi virus in *Sorex isodon*, respectively. A phylogenetic analysis of the available diversity of hantaviruses reveals the existence of four phylogroups that infect a range of mammalian hosts, as well as the occurrence of ancient reassortment events between the phylogroups. Notably, the phylogenetic histories of the viruses are not always congruent with those of their hosts, suggesting that cross-species transmission has played a major role during hantavirus evolution and at all taxonomic levels, although we also noted some evidence for virus-host co-divergence. Our phylogenetic analysis also suggests that hantaviruses might have first appeared in Chiroptera (bats) or Soricomorpha (moles and shrews), before emerging in rodent species. Overall, these data indicate that bats are likely to be important natural reservoir hosts of hantaviruses.

## Author Summary

Hantaviruses are important human pathogens, occasionally emerging from animal reservoirs. However, both the biodiversity of hantaviruses in nature, as well as the frequency with which they have jumped species barriers in the past, are unclear. Here, we describe four novel hantaviruses (Huangpi virus, Lianghe virus, Longquan virus, and Yakeshi virus) that were sampled from bats and shrews in China. These viruses are different from known hantaviruses, with each representing a novel species. An evolutionary analysis of all known hantaviruses including the novel viruses described here reveals the existence of four distinct phylogenetic groups of viruses that infect a range of mammalian hosts, and which have sometimes exchanged genes through segment reassortment. Our analysis also suggests that hantaviruses might have first appeared in bats or insectivores, before spreading to rodents, even though rodents are currently the best documented hosts of hantaviruses. Because the phylogenetic trees of the hantaviruses do not always match those of their mammalian hosts, we conclude that both host-jumping and co-divergence have played important roles in hantavirus evolution. Overall, our study shows that bats are likely to be important natural reservoir hosts of hantaviruses from which novel hantaviruses may emerge in the future.

## Introduction

Emerging infectious diseases have a substantial and ongoing impact on public health and agricultural production [1]–[3]. Over half of the currently recognized pathogens are zoonotic, and nearly all of the most important human pathogens are either zoonotic or originated as zoonoses before adapting to human transmission [4], [5]. Hence, wildlife species play a key role in disease emergence by providing a “zoonotic pool” from which previously unknown pathogens may emerge [1]. A major goal of infectious disease research is therefore to characterize those unknown pathogens circulating in animal host reservoirs before they emerge in human populations [6], [7].

Hantaviruses (genus *Hantavirus*, family *Bunyaviridae*) are the etiological agent(s) of hemorrhagic fever with renal syndrome (HFRS) and hantavirus pulmonary syndrome (HPS) in humans [8]. Unlike the other genera of *Bunyaviridae*, hantaviruses are not known to be transmitted by arthropods, and instead are harbored by small mammals, particularly rodents [9]. The first hantavirus (Thottapalayam virus (TPMV)), was isolated from the Asian house shrew (*Suncus murinus*) in India in 1964 [10], but it had not been classified as a Bunyavirus until 1989 [11]. All hantaviruses found subsequently and until 2006 were from Muroidea (i.e. ‘mouse-like’) rodents. To date, only rodent-borne viruses have been shown to cause human diseases, namely HFRS in Eurasia and HPS in the Americas [8]. As the phylogeny of the rodent-borne hantaviruses appears to be largely congruent with that of subfamily Muridae and family Cricetidae of Muroidea, hantaviruses are often considered to have co-diverged with their rodents hosts over time-scales of millions of years [12]–[15].

Since 2006, at least 22 new species of hantaviruses have been identified in Soricomorpha insectivores (shrews and moles) worldwide [9], [16]. Recently, TPMV was also found in China, Nepal, and Vietnam [17]–[19], and is thought to have had an early evolutionary divergence from rodent-borne hantaviruses [20], [21]. More recently, hantavirus RNA sequences have been detected in bats from western Africa [22], [23]. The presence of newly described hantaviruses in insectivores and bats has challenged the conventional view that hantaviruses originated from rodents, and suggests there may be additional unrecognized hantaviruses circulating in a wide range of animal hosts. Furthermore, that the viruses sampled from rodents and insectivores (Soricomorpha) do not form strict monophyletic groups [22], [24], [25] indicates that host jumping has also occurred during the evolutionary history of these viruses. As a consequence, the respective roles of virus-host co-divergence and cross-species virus transmission are more complex than previously envisioned, although determining the relative frequency of these two processes is critical for understanding the evolutionary and biogeographic processes that have produced the current diversity of hantaviruses and their potential for future emergence.

In this study, we describe four novel hantavirus sequences detected in bats and shrews collected in China. With these data we then explore key aspects of hantavirus evolution, particularly the frequency of cross-species virus transmission.

## Results

### Collection of bats and insectivores, and the detection of hantavirus RNA

**Figure 1:**
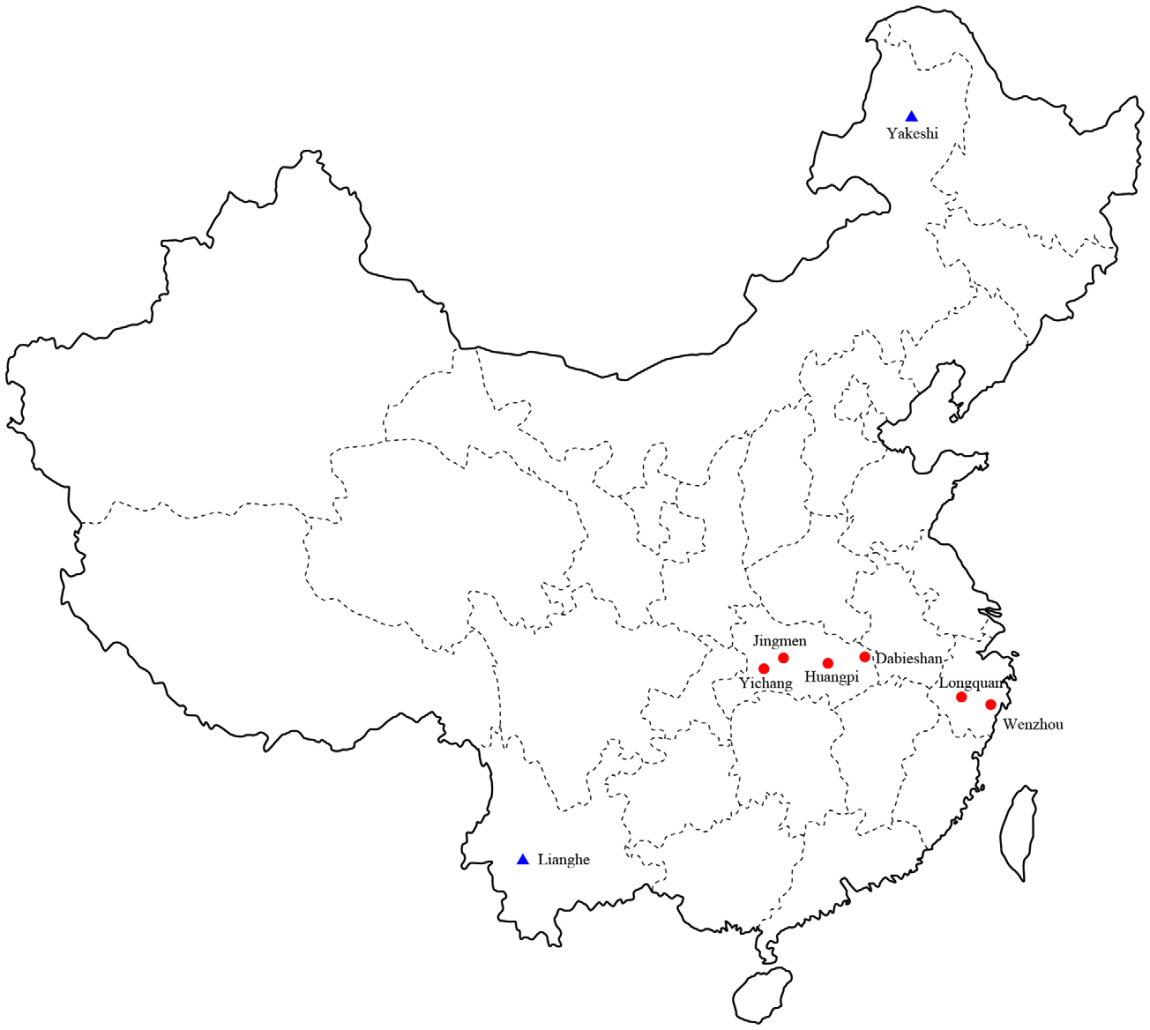
A map of China illustrating the location of trap sites in which bats (red circular) and shrews (blue triangle) were captured.

A total of 450 bats of eight different species were captured in Longquan city and Wenzhou city, Zhejiang Province in the spring of 2011 (Figure 1 and Table 1). Similarly, 155 bats representing eight species were captured in Hubei Province in the spring of 2012. A total of 81 insectivores (representing two species – *Anourosorex squamipes* and *Suncus murinus*) were captured in Lianghe county, Yunnan Province in the spring of 2010 and autumn of 2011. In 2006, two shrews (from the species *Sorex isodon* and *Suncus murinus*) were collected from Yakeshi city, Inner Mongolia Autonomous Region.

**Table 1:** Prevalence of hantavirus in bats and insectivores by species and location in China.

Species	Zhejiang		Hubei				Inner Mongolia	Yunnan	Total
	Longquan	Wenzhou	Yichang	Jingmen	Huangpi	Dabieshan	Yakeshi	Lianghe	
Bats									
*Rhinolophus pearsonill*	0/29	-	-	-	-	-	-	-	0/29
*Rhinolophus monoceros*	1/3	0/1	-	-	-	-	-	-	1/4
*Rhinolophus affinis*	6/23	0/3	-	-	-	-	-	-	6/26
*Rhinolophus sinicus*	3/133	0/2	-	-	-	-	-	-	3/135
*Rhinolophus pusillus*	0/1	0/235	-	0/14	-	-	-	-	0/250
*Rhinolophus macrotis*	-	-	-	0/7	-	-	-	-	0/7
*Rhinolophus ferrumequinum*	-	-	-	0/9	-	-	-	-	0/9
*Hipposideros armiger*	-	0/7							0/7
*Ia io*	-		0/1	-	-	-	-	-	0/1
*Miniopterus schreibersii*	-	0/9	-	0/2	-	-	-	-	0/11
*Myotis chinensis*	-	0/3							0/3
*Myotis altarium*	-	-	0/26	-	-	-	-	-	0/26
*Murina leucogaster*	-	-	0/92	-	-	-	-	-	0/92
*Pipistrellus abramus*	-	0/1	-	-	1/3	0/1	-	-	1/5
Sub-total	10/189	0/261	0/119	0/32	1/3	0/1	-	-	11/605
Insectivores									
*Sorex isodon*	-	-	-	-	-	-	1/2	-	1/2
*Anourosorex squamipes*	-	-	-	-	-	-	-	9/59	9/59
*Suncus murinus*	-	-	-	-	-	-	0/3	0/19	0/22
Sub-total	-	-	-	-	-	-	1/5	0/78	10/83

Note: “-” means that no animals were captured.

RT-PCR was performed to detect hantaviral RNA based on the L segment sequences. In bats, PCR products of the expected size were amplified from six *Rhinolophus affinis*, three *Rhinolophus sinicus*, one *Rhinolophus monoceros* collected from Longquan, and one *Pipistrellus abramus* from Huangpi. In insectivores, expected size products were generated from one *Sorex isodon* from Yakeshi and nine *Anourosorex squamipes* from Lianghe. These sequences most closely resembled those of other hantaviruses (Table S1) (see below).

### Characterization of viral sequences

To characterize the novel hantaviruses found in this study, sequences of the complete S and M segments were recovered from the RNA positive bat and shrew samples described above. Key features of these sequences are described in detail in Table 2 and Figure S1. Clearly, the viruses from bats and shrews are distinct from each other and from other known hantaviruses, representing four novel species of hantavirus (see below). We therefore named these new viruses as Huangpi virus (HUPV), Longquan virus (LQUV), Lianghe virus (LHEV), and Yakeshi virus (YKSV), and which were found in *P. abramus*, *Rhinolophus spp.* (*R. affinis*, *R. sinicus*, and *R. monoceros*), *A. squamipes*, and in *S. isodon*, respectively. HUPV, LQUV, and YKSV exhibit ≤89.6% sequence similarity in the N, GPC and L proteins from all known hantaviruses (Tables S2, S3). In contrast, LHEV is clearly related to Cao Bang virus (CBNV) also identified in *Anourosorex squamipes* in Vietnam [26] in sequences of the N (≤95.6% similarity), GPC (≤92.7%) and L (≤94.3%) proteins. However, LHEV is different from CBNV in the GPC protein, exhibiting more than the 7% amino acid difference required for hantavirus species demarcation [9].

**Table 2:** The structure of the hantavirus S and M segments.

Virus	Segment	Length (nt)	5′ NCR (nt)	ORF (nt)	Protein (AA)	3′ NCR (nt)	Position of NS	NS(AA)
LQUV	S	1545–1568	54	1272	423	219–242	N	N
	M	3618–3622	20	3402	1133	196–200	N	N
HUPV(partial)	S	1115	-	816	271	299	-	-
	M	-	-	-	-	-	-	-
LHEV	S	1804–1814	38	1287	428	479–489	N	N
	M	3628–3632	40	3420	1139	168–172	N	N
YKSV	S	1686	46	1290	429	350	N	N
	M	3460	40	3420	1139	170	N	N
TPMV	S	1530	67	1308	435	155	N	N
	M	3621	40	3405	1134	216	N	N
NVAV	S	1839	52	1287	428	500	N	N
	M	-	-	-	-	-	-	-
Murinae (L99)	S	1746	42	1290	429	432	N	N
	M	3652	46	3402	1133	204	N	N
Arvicolinae (DTK/Ufa-97)	S	1829	43	1302	433	484	84–356	90
	M	3682	40	3447	1148	195	N	N
Sigmodontinae (Chile-9717869)	S	1871	42	1287	428	542	122–313	63
	M	3671	51	3417	1138	203	N	N

### Phylogenetic relationships among the novel and known hantaviruses

**Figure 2:**
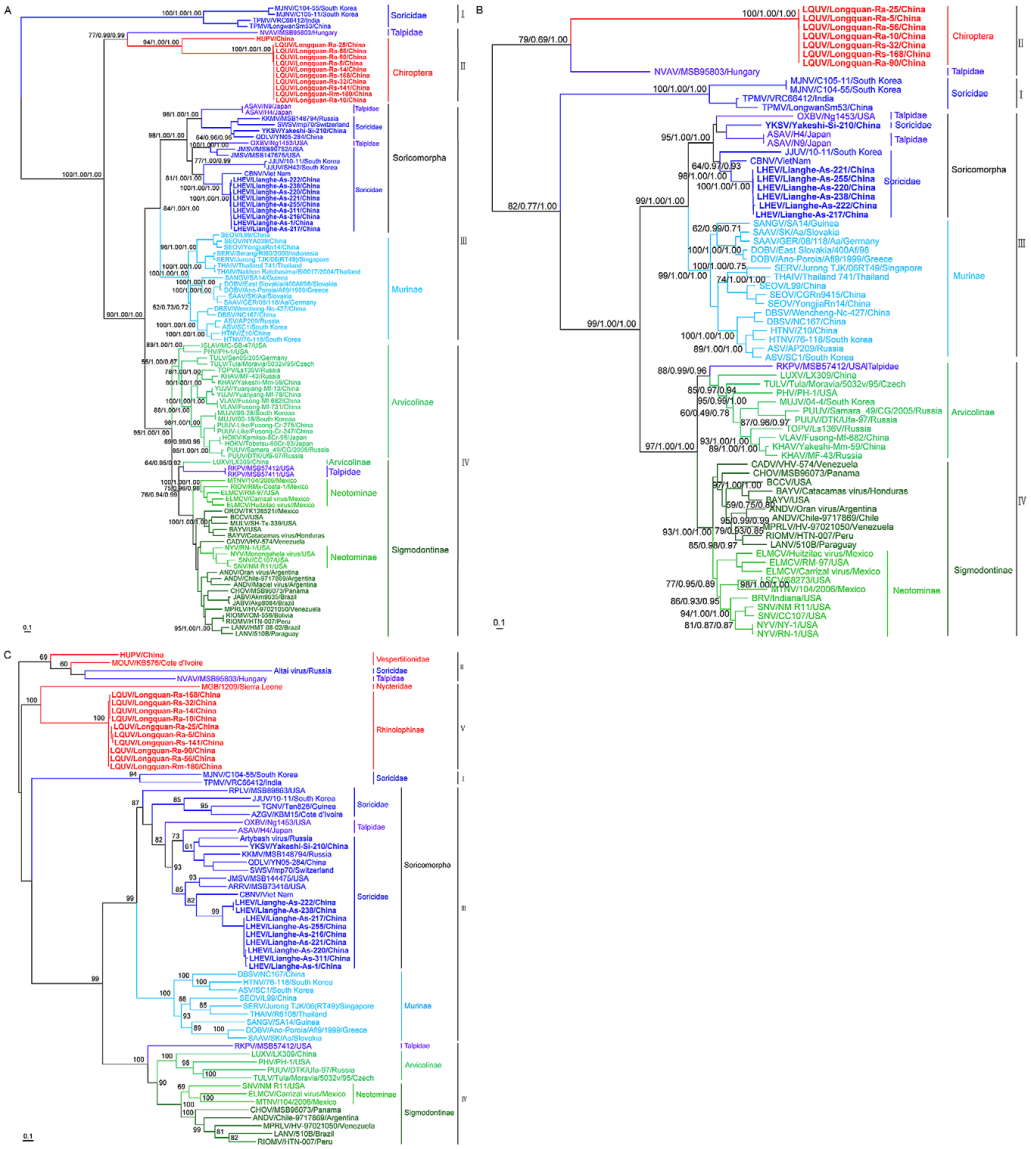
Phylogenetic trees based on the entire coding regions of hantavirus genomes including those obtained here inferred using the BEAST (MCC), Bayesian, and ML methods. The ML/MCC/Bayesian trees were based on the coding sequences of the S (A) and M (B) segments. Numbers (>70%/>0.7/>0.7) above or below branches indicate posterior node probabilities or bootstrap values. Phylogenetic tree (C) was inferred using the ML method based on the partial L segment sequences. The MCC tree – shown here in all cases – was automatically rooted on the assumption of a molecular clock. The basal lineage estimated by the MCC tree was then used as an outgroup in the Bayesian and ML analyses. Scale bar represents number of nucleotide substitutions per site.

To determine the phylogenetic relationships among the novel hantaviruses described here and those described previously, phylogenetic trees based on 103 S and 71 M segment sequences were inferred using three methods. The Bayesian and Maximum Likelihood (ML) trees were rooted in the way suggested by the (molecular clock-rooted) MCC tree. The ML trees based on the M or S segment sequences produced very similar topologies (Figure 2). In the S segment tree (Figure 2A), all known hantaviruses including the viruses identified in bats and insectivores could be placed into four well supported ‘phylogroups’. The first phylogroup only comprised viruses from insectivore (Soricidae) species and included the Asian viruses TPMV and Imjin virus (MJNV) sampled from the Ussuri white-toothed shrew (*Crocidura lasiura*) in South Korea [27]. Notably, this phylogroup occupied a basal position with respect to the remaining viruses. The second phylogroup comprised HUPV and LQUV found in bats in this study and which were closely related each other, along with the more divergent Nova virus (NVAV) identified in the European common mole (*Talpa europaea*) in Hungary [24]. Phylogroup III contained all other known Soricomorpha-associated viruses, including LHEV and YKSV found in this study, as well as a distinct clade of Murinae-borne (i.e. rodent) viruses. Finally, the fourth phylogroup included viruses sampled from the Arvicolinae, Neotominae, and Sigmodontinae subfamilies of rodents, although these did not form three clearly distinct monophyletic groups in the S segment, along with the reassortant RKPV sampled from an insectivore (see below). Importantly, the topologies of ML and Bayesian trees estimated using amino acid sequences of N (encoded by the S segment) and GPC (encoded by the M segment) proteins were consistent with those of the trees based on the nucleotide sequences, indicating that site saturation has not adversely affected our phylogenetic inference (Figure S2A–F). Although a closer phylogenetic relationship between the first and second phylogroups were observed in the Bayesian tree (Figures S2B and S2E), these two phylogroups still occupied basal positions.

The most striking difference between the S and M segment trees was that phylogroup II (i.e. LQUV and NVAV) were basal in the M segment tree with relatively strong statistical support (Figure 2B) (although it is important to note that we were unable to amplify the M segment sequence from HUPV), while the Soricidae-associated viruses of phylogroup I occupied the basal position in the S segment tree and with much stronger support (Figure 2A). This different phylogenetic pattern was also apparent in the relevant amino acid trees of the N and GPC proteins (Figure S2A–F). Such phylogenetic incongruence is strongly suggestive of reassortment among hantaviruses of phylogroups I and II, and which might have occurred during the evolution of hantaviruses carried by bats and insectivores as these phylogroups are currently only associated with these mammalian species. Irrespective of this history of reassortment it is clear that there have been multiple cross-species transmission events in the evolutionary history of the hantaviruses with, for example, those viruses sampled Soricomorpha forming a paraphyletic group, as do those from bats shown in the L tree.

In both the M and S segment trees YKSV (a member of the Soricomorpha clade of phylogroup III) showed a close phylogenetic relationship with Qiandao lake virus (QDLV) sampled from *Sorex cylindricauda* in China (GU566023), Kenkeme virus (KKMV) collected from the Flat-Skulled Shrew (*Sorex roboratus*) in the far eastern Asian region of Russia [28], Seewis virus (SWSV) from the Eurasian common shrew (*Sorex araneu*s) in Switzerland [29], and Asama virus (ASAV) from the Japanese shrew mole in Japan (*Urotrichus talpoides*) [30]. Hence, this well-supported subgroup contained four viruses from Asia and one from Europe. Also of note was that all LHEV sequences exhibited a close relationship with CBNV and Jeju virus (JJUV) sampled from the Asian lesser white-toothed shrew (*Crocidura shantungensis*) in South Korea [16], Jemez Springs virus (JMSV) from the dusky shrew (*Sorex monticolus*) [31] and Oxbow virus (OXBV) from the American shrew mole (*Neurotrichus gibbsii*) [32], with the latter two viruses both found in the USA.

Kang et al. [33] found that RKPV sampled from a *S. aquaticus* mole in the USA shared a closer relationship with viruses harbored by Cricetidae rodents than with Soricomorpha-borne hantaviruses, a topology confirmed by our analysis. Interestingly, in the S segment tree RKPV was most closely related to another novel hantavirus (LUXV) identified in the Yunnan red-backed vole (*E. miletus*) in China [34]. More notable was that both viruses were more closely related to Sigmodontinae/Neotominae-borne hantaviruses in the S segment tree but with Arvicolinae-borne hantaviruses in the M segment tree, suggesting that both LUXV and RKPV were generated by a common reassortant event (Figures 2A–2B).

A rather different picture of the evolutionary history of hantaviruses was observed in the phylogenies of 62 L segment sequences. In particular, these trees provided evidence for five phylogroups, as viruses from phylogroup II could be subdivided into a subgroup containing HPUV, Mouyassué virus (MOUV) detected in bat from Cote d'Ivoire [22], NVAV, and Altai virus (EU424341) sampled from a Soricidae shrew in the neighboring area of Russia with China, and a subgroup containing the LQUV and MGB virus sampled from bats in Sierra Leone [23] (phylogroup V, Figure 2C). However, this novel subdivision of phylogroups was not supported strongly. The clustering patterns of other viruses were similar to those in the S and M segment trees (Figure S3A–B), although LQUV and MGB virus grouped with TPMV and MJNV in the Bayesian tree (Figure S3B). Finally, and in contrast what is seen in the L nucleotide sequence phylogenies, MGB virus shared a closer relationship with TPMV and MJNV than HUPV and LQUV in the L amino acid tree (Figure S2G–I).

### Geographic distribution of hantaviruses

**Figure 3:**
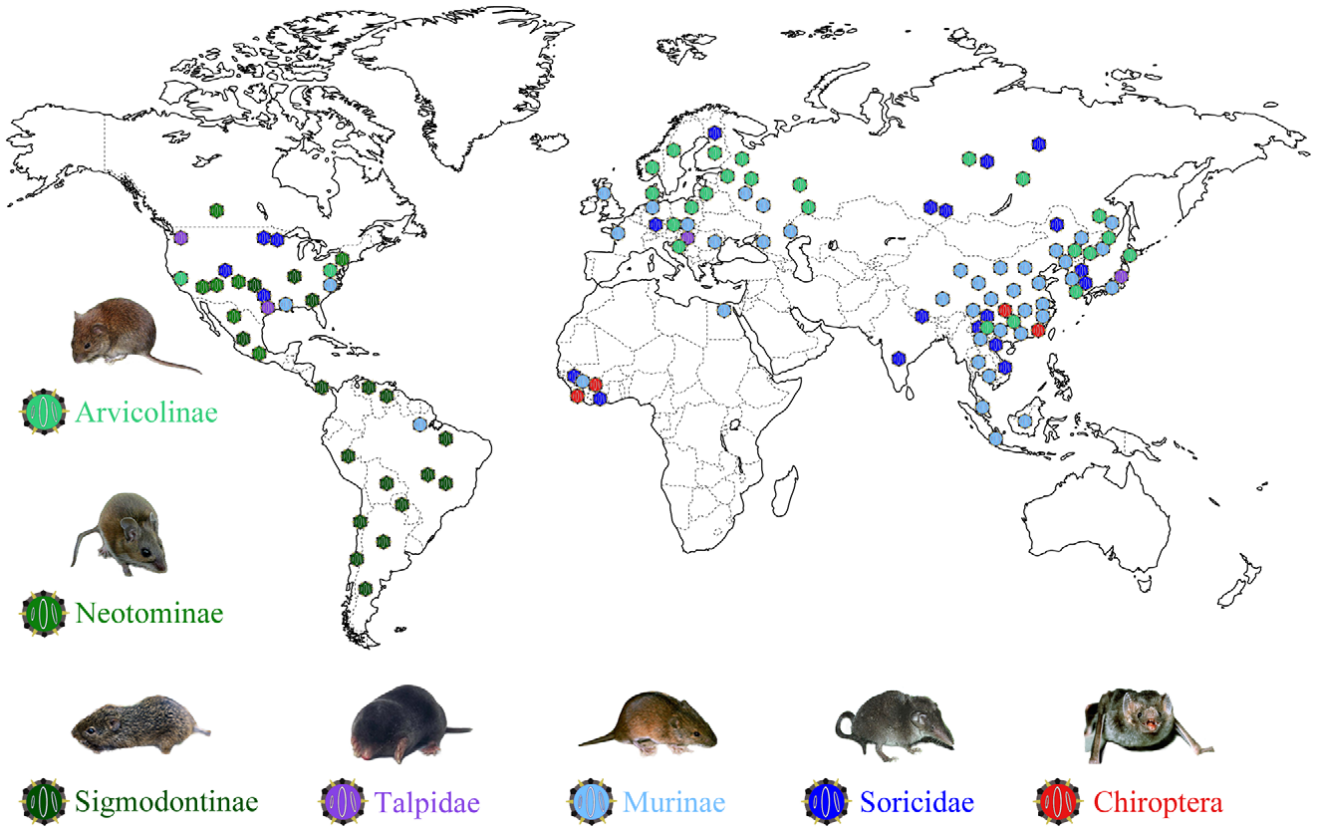
A map of the world illustrating the location of known hantaviruses by host group and associated mammalian hosts.

Our phylogenetic analysis also provided insights into the geographic distribution of these viruses. All those S segment phylogroup I viruses identified so far are from Asia (Soricidae, Figure 3), while phylogroup II viruses have been recovered from both Asia and Europe (Talpidae and Chiroptera). In the L gene tree the two viruses found in African bats were closely related to HPUV and LQUV found in bats from China, respectively (Figure 2C). With respect to phylogroup III, viruses of Soricomorpha clade have been mainly found in Asia, with a few from Europe, North America, and Africa. With the exception of Sangassou virus (SANGV) found in the wood mice from Guinea [35], almost all viruses of the Murinae clade are from Asia and Europe. Finally, for phylogroup IV, most of the Arvicolinae clade viruses have been identified in Asia and Europe, with a few sampled from North American animals. In contrast, almost all viruses of the Sigmodontinae clade are from the New World, and the lineage comprising LUXV from China and RPKV found in USA occupied a basal position in this clade. Overall, those hantaviruses sampled from Asian mammalian species exhibit the greatest genetic diversity and tend to fall at basal positions on the phylogenetic trees. This tentatively suggests that hantaviruses may have an Asian origin, although this will need to be confirmed on a far larger sample of taxa.

### Co-divergence and cross-species transmission in hantavirus evolution

**Figure 4:**
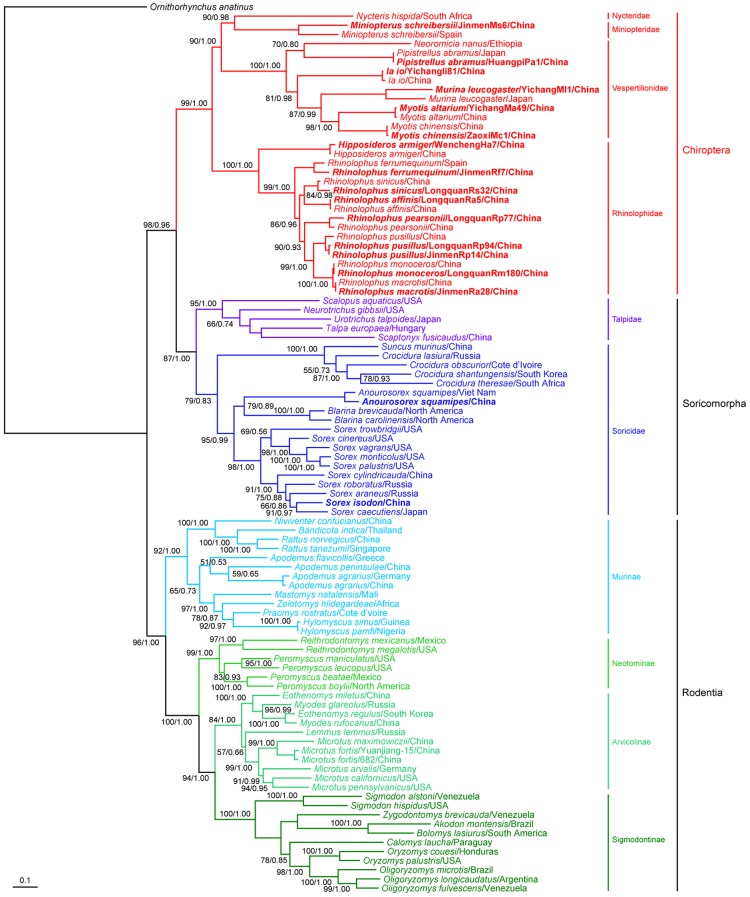
Phylogenetic relationships between bats, insectivores, and rodents captured in China and others taken from the GenBank. The ML and MCC trees were constructed with the mt-*cyt b* gene. The sequences of *Ornithorhynchus anatinus* were used as an outgroup. The sequences obtained in this study are shown in bold. Posterior node probabilities (70%/>0.7) are shown above or below branches. Scale bar represents number of nucleotide substitutions per site.

We inferred ML and MCC trees of mitochondrial cytochrome *b* (mt-*cyt b*) gene sequences among the known mammalian hosts (Chiroptera, Soricomorpha, and Rodentia) of the hantaviruses. Both trees had very similar topologies. Specifically, using *Ornithorhynchus anatinus* as an outgroup, the rooted phylogenetic trees based on mt-*cyt b* gene sequences including the sequences obtained in this study (Table S4) resulted in a clear phylogenetic division between those viruses sampled from Rodentia, Chiroptera and Soricomorpha, with each forming a monophyletic group as expected (Figure 4). In agreement with previous studies [36], Soricomorpha showed a closer relationship with Chiroptera than with Rodentia. Within Rodentia, the Murinae subfamily and Cricetidae family formed two monophyletic groups. The Cricetidae were further subdivided into the subfamilies Neotominae, Arvicolinae and Sigmodontinae. Based on this single-locus study, Neotominae, which was once considered an exclusively North American subset of the South American Sigmodontinae, was more closely related to Arvicolinae than Sigmodontinae. However, studies based on multiple nuclear loci place the Neotiminae as a distinct sister subfamily with the Sigmodontinae [37].

**Figure 5:**
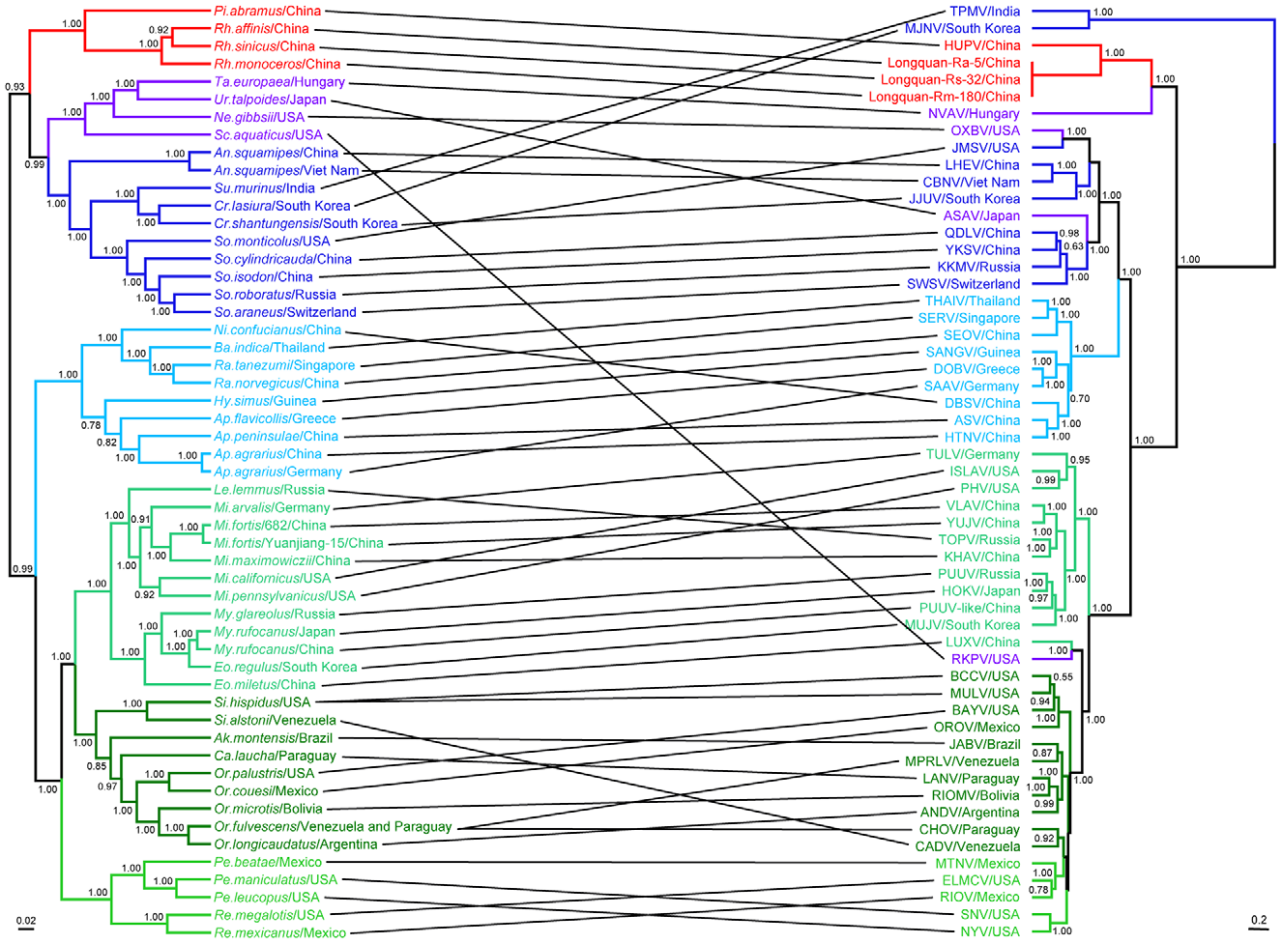
Tanglegram comparing the phylogenies of hantaviruses and their bat, insectivore, and rodent hosts. The host tree on the left was based on cytochrome b gene sequences, while the hantavirus tree on the right was based on the coding sequences of S segment. Numbers (>0.7) above or below branches indicate posterior node probabilities.

We used TreeMap 2.0 to test the strength of congruence between the viral S, M, and S+M segment trees with that of the host mt-*cyt b* gene (Figure 5, Figure S4; Table S5). Notably, the viral phylogenies inferred using the S segment sequences were not always consistent with their hosts' phylogeny as measured by both CE (*P* = 0.098±0.009) and NCE (*P* = 0.1±0.009) frequencies, with multiple deep and more recent topological differences, and hence an indication of relatively frequent host jumping (Figure 5). This analysis also indicated that cross-species transmission events had occurred at four levels during hantavirus evolution (Figure 5, Table 3): inter-species within a genus (e.g. HTNV and ASV, DOBV and SAAV), inter-genus within a family (e.g. DBSV and HTNV), inter-family within an order (e.g. OXBV and JMSV, ASAV and SWSV), and even inter-order (e.g. NVAV and LQUV; LUXV and RKPV). In addition, some viruses exhibited a phylogenetic pattern that reflected their geographic origins rather than the phylogeny of their hosts – such as viruses OXBV and JMSV, DOBV and SAAV within the Soricomorpha and Murinae clades of phylogroup III (Table S5) – such that the likelihood of host jumping in part reflects geographic proximity. However, in other instances there were clear matches between the virus and host phylogenies. Most notably, there was significant congruence between phylogenies of the two clades of phylogroup IV and their rodent hosts - Arvicolinae (CE (*P* = 0.006±0.002) and NCE (*P* = 0.005±0.002)) and Sigmodontinae (CE (*P* = 0.041±0.006) and NCE (*P* = 0.01±0.003)) – indicating that these rodent hantaviruses may have a long history in their primary hosts, likely co-diverging with their hosts in some cases.

**Table 3:** Inferred cross-species transmission events among the hantaviruses.

Host		Virus			
Species	Genetic distance	Virus pairs	^+^ (%) nt and aa similarities between virus pair		Level
			S	M	
*Talpa europaea - Rhinolophus spp.*	0.241	NVAV-LQUV	55.3/55.1	-	Order
*Eothenomys miletus - Scalopus aquaticus*	0.165	LUXV-RKPV	72.7/80.8	65.2/62.4	Order
*Neurotrichus gibbsii - So. monticolus*	0.227	OXBV-JMSV	74.8/84.3	-	Family
*Urotrichus talpoides - So. araneus*	0.204	ASAV-SWSV	63.5/69.9	-	Family
*Suncus murinus - Crocidura lasiura*	0.162	TPMV-MJNV	65.5/70.6	68.2/71.9	Genus
*Cr. shantungensis - Anourosorex squamipes*	0.214	JJUV - CBNV	69.6/70.3	-	Genus
*Hylomyscus simus - Apodemus flavicllis*	0.119	SANGV-DOBV	78.2/88.1	73.9/81.8	Genus
*Bandicota indica - Rattus rattus*	0.123	THAIV-SERV	84.1/97.2	-	Genus
*Niviventer confucianus - Ap. agrarius*	0.173	DBSV-HTNV	78.2/92.1	76.2/84.7	Genus
*Microtus fortis - Lemmus sibiricus*	0.149	KHAV-TOPV	82.5/95.6	77.6/88.6	Genus
*Oligoryzomys palustri - Sigmodon. hispidus*	0.186	BAYV- BCCV	81.9/92.3	77.7/88.5	Genus
*Ol. fulvescens - Akodon montensis*	0.198	MPRLV - JABV	79.0/89.3	-	Genus
*Ol. microtis - Calomys laucha*	0.178	RIOMV-LANV	82.4/93.2	78.5/91.2	Genus
*Peromyscus beatae - Reithrodontomys megalotis*	0.159	MTNV-ELMCV	78.7/91.6	73.4/80.7	Genus
*Ap. flavicllis - Ap. agrarius*	0.159	DOBV-SAAV	87.3/98.1	83.0/94.2	Species
*Ap. agrarius - Ap. peninsulae*	0.154	HTNV-ASV	84.1/97.0	80.5/91.7	Species
*Myodes rufocanus - My. glareolus*	0.101	HOKV-PUUV	82.6/94.9	-	Species

Note: The cross-species transmission events listed in this table were inferred by comparing the host (mt-*cyt b*) and virus (S, M, and S+M segment) phylogenies and looking for patterns of incongruence (Figure 5, Figure S4; Table S5).

nt (nucleotide); aa (amino acid).

+Compared with other hantaviruses, virus pairs shared the highest similarities in the nt and aa sequences of their S and M segments.

## Discussion

We describe four novel hantavirus sequences from bats and insectivores captured in China. The hantavirus harbored by three *Rhinolophus* bats and one carried by the *Sorex isodon* shrew exhibited ≤89.6% amino acid similarity in the N, GPC and L protein sequences with any recognized hantaviruses, while the hantavirus carried by one *Pipistrellus* bat shared ≤81.9% amino acid similarity in both the N and L protein sequences with known hantaviruses. The hantavirus found in *Anourosorex squamipes* (shrew) from Lianghe (Yunnan Province) was most closely related to CBNV also identified in *Anourosorex squamipes* in Vietnam, but with quite different N (>4.4% amino acid), L (>5.7%), and GPC (>7.3%) amino acid sequences. Interestingly, the mt-*cyt b* gene differences between *Anourosorex squamipes* in Yunnan of China and Vietnam are 1.7%, compatible with the existence of the two subspecies of *Anourosorex squamipes*. According to the criteria for species demarcation in the genus *Hantavirus* proposed by the International Committee on Taxonomy of Viruses [9], these four hantaviruses are sufficiently genetically distinct that they should be recognized as distinct species. Accordingly, we propose naming these four novel hantaviruses as Huangpi virus (HUPV), Lianghe virus (LHEV) Longquan virus (LQUV), and Yakeshi virus (YKSV), reflecting their geographic origins. In addition, as LHEV has not been isolated, such that two-way cross neutralization tests cannot be performed, further studies are needed to clarify whether LHEV is a novel species or simply a variant of CBNV. Finally, the identification of LQUV in three *Rhinolophus* bats also means that hantaviruses may spread relatively easily among different species of bats.

Although rodents are considered the primary hosts of hantaviruses [13], the increasing number of viruses found in insectivore species (shrews and moles) over the past five years has raised an important question mark over the host range and origin of hantaviruses. Indeed, the first hantavirus (TPMV) was isolated from shrews in India in 1964 [10]. Our work further suggests that bats are likely to be important hosts for hantaviruses. Bats (order Chiroptera) have been shown to be sources of a broad variety of emerging pathogens, including coronaviruses, filoviruses, henipaviruses, and lyssaviruses [38]. Recently, partial hantaviral sequences were found in one slit-faced bat (*Nycteris hispida*) and two banana pipistrelles (*Neoromicia nanus*) in West Africa [22], [23]. We document two novel hantaviruses in *Rhinolophus* bats (*R. affinis, R. sinicus*, *R. monoceros*) and *P. abramus*. Consequently, these data, together with other recent studies [22], [23], demonstrate that bats in China and Africa are hosts of hantaviruses and thereby constitute a potential sylvatic mammalian reservoir of hantaviruses. As their global distribution, abundance, ability to fly long distances, often large population densities, and sociality favor the efficient maintenance, evolution, and spread of viruses, it is clear that further study is needed to elucidate the potential importance of bats as hantavirus hosts. Indeed, it seems likely that additional hantaviruses will be isolated from bats, and especially from insectivorous bats as all four bat species in which hantavirus sequences were detected in this study are insectivorous. Moreover, because they consistently occupy basal phylogenetic positions, these phylogenetic data suggest that the ancestor of the extant hantaviruses might have first appeared in Chiroptera and/or Soricomorpha, although this will need to be confirmed on a larger sample of mammalian taxa.

One notable feature of our phylogenetic analysis was the basal position of phylogroup I viruses in the S segment tree but of phylogroup II viruses in the M segment. Such deep phylogenetic incongruence is strongly suggestive of an ancient reassortment event. In the S segment tree, HUPV and LQUV share a closer relationship with NVAV identified in *Talpa europaea* [24], as does LQUV in the M segment tree and HPUV in the S segment tree, suggesting that these viruses share common ancestry. In addition, hantaviruses identified in bats in Africa are closely related to NVAV or TPMV [22], [23]. Within phylogroup IV, RKPV identified in a mole is closely related to LUXV from the Yunnan red-backed vole in China. These viruses also share a history of reassortment since they occupy the basal positions within the Arvicolinae clade in the M segment tree but with the Sigmodontinae clade in the S segment tree.

The current geographic distribution of hantaviruses in large part reflects that of their host species [13], [39]. If hantaviruses have indeed been associated with mammalian species for millions of years, then it is possible that these geographic distributions are long established. The oldest eutherian is *Juramaia sinensis* (an insectivore) found in China, at an estimated 160 million years ago [40]. It was recently suggested that both Euarchontoglires and Laurasiatheria, excluding Chiroptera, originated in Eurasia [41]. Geographic reconstructions further suggest that bats originated in the Laurasian land masses, with an Asian origin for the suborder Yinpterochiroptera and a most likely Asian/European origin for the suborder Yangochiroptera [42]. Within the insectivores, Talpidae occupies the basal position within the Soricomorpha [36], [43], and both molecular clock dating and the fossil record suggest a Eurasian origin of the Soricidae [43]–[45]. As the hantaviruses sampled from Asian mammals are genetically very diverse and tend to occupy basal positions in the phylogenetic trees, these data tentatively support an Asian origin for hantaviruses, although this will need to be assessed on a more geographically diverse sample.

Hantaviruses have traditionally been considered to have co-diverged (including co-speciation) with their rodent hosts on time-scales of millions of years [12]–[15], and some evidence for such co-divergence was apparent here. In particular, rodent hantaviruses clustered according to whether their hosts were members of the Murinae subfamily and Cricetidae family. Indeed, the close phylogenetic relationships among some hantavirus taxa across large geographic areas, and which infect related hosts, supports the occurrence of long-term virus-host co-divergence [46]. Hence, rodent hantaviruses might have a long history in their primary hosts, and which in part explains their biodiversity [12]. Despite this, more examples of incongruence between the gene trees of hantaviruses and their hosts are being identified, suggesting that some of the congruence between the two might have arisen from preferential host switching and local adaptation [25]. Indeed, it was recently shown that cross-species transmission has even played a role in shaping the genetic diversity of the currently known Murinae-associated hantaviruses [46]. In accord with this, the current study provides evidence for cross-species transmission events at the family, genus, and species levels. In particular, that viruses from both the Chiroptera and Soricomorpha form paraphyletic groups in all our analyses strongly suggests that ancestral hantaviruses jumped between mammalian orders. As a consequence, it is clear that cross-species virus transmission as well as the geographic dispersal of Chiroptera, Soricomorpha and Rodentia, has also contributed to the high biodiversity and near global distribution of those hantaviruses known today, although the time-scale of these host jumping events remains uncertain.

## Materials and Methods

### Ethics statement

This study was reviewed and approved by the ethics committee of the National Institute for Communicable Disease Control and Prevention of the Chinese CDC. All animals were treated in strict according to the guidelines for the Laboratory Animal Use and Care from the Chinese CDC and the Rules for the Implementation of Laboratory Animal Medicine (1998) from the Ministry of Health, China, under the protocols approved by the National Institute for Communicable Disease Control and Prevention. All surgery was performed under ether anesthesia, and all efforts were made to minimize suffering.

### Trapping of small animals and specimen collection

Bats were captured with mist nets or harp traps in caves of natural roosts in Zhejiang Province in the spring of 2011, or in villages or caves in Hubei Province in the spring of 2012 (Figure 1). According to protocols described previously [47], insectivore animals were trapped in cages using fried foods as bait in the Inner Mongolia Autonomous Region in 2006 or in Yunnan Province in the autumns of 2010 and 2011. All animals kept were alive after capture. They were initially identified by morphological examination according to the criteria for bats described by Wang [48] and for insectivores by Chen [49], and further confirmed by sequence analysis of the mt-*cyt b* gene. All animals were anesthetized with ether before surgery, and all efforts were made to minimize suffering. Tissue samples of heart, liver, spleen, lung, kidney and brain were collected from bats and insectivores for detecting hantaviruses.

### DNA and RNA extraction, PCR and sequencing

Total DNA was extracted using the DNeasy Blood & Tissue kit (QIAGEN) from tissue samples of bats or insectivores according to the manufacturer's protocol. The mitochondrial (mt)-*cyt b* gene (1140 bp) was amplified by PCR with the primer pair for bats [50] and one for insectivores [51].

Total RNA was extracted from tissue samples using TRIzol reagent (Invitrogen, Carlsbad, CA) according to the manufacturer's instructions. cDNA was prepared with AMV reverse transcriptase (Promega, Beijing) with the primer P14 [52]. Hantaviral RNA was detected by RT-PCR as described previously [17], [35]. Primers designed based on the conserved regions of known complete S and M segment sequences from hantaviruses were used to amplify the entire S and M segments. In the amplification of the 5′ terminus of unknown hantaviruses, an adaptor plus P14 was used as a primer in the synthesis of cDNA. Semi-PCR was used to amplify the 5′ terminus with the adaptor as the forward primer and two specific reverse primers. Semi-PCR was also used to amplify the 3′ terminus with two specific forward primers and an adaptor plus modified P14 (5′-TAGTAGTRGACWCC-3′) [52] as the reverse primer. Primer sequences used in this study are provided in Table S6. The RT-PCR products were separated by agarose gel and further purified using the Agarose Gel DNA Purification kit (TaKaRa, Dalian, China). Amplicons less than 700 bp were sequenced from both directions. Amplicons greater than 700 bp were cloned into pMD18-T vector (TaKaRa, Dalian, China). Sequencing was performed using the ABI-PRISM Dye Termination Sequencing kit and ABI 373-A genetic analyzer. At least three clones were sequenced.

### Sequence data

One to three sequences of the entire open reading frame (ORF) were randomly chosen from each hantavirus species for phylogenetic analysis. The RDP3 program [53] was used to examine potential intra-segment recombination in the viral sequences, although no recombinant sequences were identified (although we do find evidence for segment reassortment – see below). Identical sequences were excluded from this study.

Both animal mt-*cyt b* gene and viral genome sequences were aligned using the ClustalW method implemented in the Lasergene program, version 5 (DNASTAR, Inc., Madison, WI). Poorly aligned positions and divergent regions of the alignment, and which could negatively affect phylogenetic analysis, were removed using Gblocks [54]. The following data set sizes were used in the final analysis: hantavirus S segment = 103 sequences, 1201 bp; M segment = 71 sequences, 3024 bp; L segment = 30 sequences, 6519 bp, partial L segment = 32, 330 bp; mt-*cyt b* gene = 97 sequences, 1140 bp.

### Phylogenetic analyses

Phylogenetic trees were estimated using the Maximum Likelihood (ML) method available at the RAxML Blackbox web-server [55]. The best-fit evolutionary model was determined using jModelTest version 0.1 [56], and found to be the General Time Reversible (GTR) with a gamma-distribution model of among site rate heterogeneity and a proportion of invariant sites (GTR+Γ+I). Phylogenetic trees were also inferred using the Bayesian method implemented in MrBayes v3.1.2 [57]. The same evolutionary model was employed as described above. For this analysis, three hot and one cold Markov chain Monte Carlo (MCMC) chains were used, sampling every 100 generations and with a 25% burn-in. The Effective Sample Size (ESS) of all parameters was larger than 200 indicating that parameter convergence had occurred.

A (molecular clock) rooted tree of these sequences was inferred using the Bayesian MCMC method available in the BEAST v1.6.0 package [58]. The same evolutionary model was employed as described above. We also incorporated a relaxed (uncorrelated lognormal) molecular clock, with an extended Bayesian Skyline tree prior. Two independent runs were undertaken sampling every 1,000 generations. Each run was continued until ESS >200 was achieved, with the output analyzed in Tracer v1.5. TreeAnnotator was used to generate a Maximum Cade Credibility (MCC) tree with a burn-in of 10% of the sampled trees. Because the MCC tree is automatically rooted on the assumption of a molecular clock it enables determination of which viral lineages are most likely to be basal. Accordingly, the basal lineage estimated by the MCC tree was used as an outgroup to root the phylogenetic trees inferred under the ML and Bayesian phylogenetic analyses. In addition, because the high levels of sequence divergence across the hantaviruses, we also inferred phylogenetic trees based on the amino acid sequences of the L protein, N protein (encoded by the S segment), and GPC protein (encoded by the M segment) using the ML approach available in the phyML program [59]. The LG amino acid substitution model was used for both the L and GPC proteins, and while the FLU model was used for the N protein.

Finally, a phylogenetic tree for the host mt-*cyt b* sequences tree was estimated using the ML and BEAST (MCC tree) methods, again employing the GTR+Γ+I substitution model as estimated by jModelTest. In the case of the BEAST analysis a relaxed (uncorrelated lognormal) molecular clock was used along with the Yule model as a coalescent prior.

To determine the degree of congruence between the phylogenies of hantaviruses and their hosts we used Tree-Map (2.0b) [60], although such analyses are complicated by uncertainties in the virus or host trees. A tanglegram was generated by matching each hantavirus species to their associated host(s). Specifically, nodes of the viral MCC tree were mapped onto the related nodes of the host (MCC) tree. Significance testing was undertaken by generating 1000 viral trees with randomized branches and mapping these random trees onto the fixed host tree. We then evaluated the proportion of these reconciliations with the same or fewer non-co-divergence events (NCEs), or the same or more co-divergence events (CEs), compared to the “real” viral tree. If the *p* value is greater than 0.05, we can reject the null hypothesis that the level of congruence is no more than that expected between randomly generated trees. Due to computational limitations in TreeMap [61], we reduced the complexity of the host and virus phylogenies as much as possible before performing the full reconciliation analysis. Thus, for viruses-host matches, we divided all hantaviruses and their hosts into four groups: (i) bats and insectivores and their viruses (Rockport virus (RKPV) was removed because it was a reassortant virus), (ii) Murinae and their viruses, (iii) Arvicolinae and their viruses (Luxi virus (LUXV) was removed because it was a reassortant virus), and (iv) Sigmodontinae and their viruses. For the full reconciliation analysis, three species (virus or host) representing each of four groups (bats and insectivores, Murinae, Arvicolinae, and Sigmodontinae) were used to compare the host and the virus phylogenies (and including *Scalopus aquaticus* and RKPV, *Eothenomys miletus* and LUXV). Cross-species transmission events were then inferred by comparing the topologies of the virus and host phylogenies. Specifically, we considered the degree of congruence between the viral S, M, and S+M segment trees with that of the host mt-*cyt b* gene tree (Figure 5, Figure S4; Table S5). Importantly, any viruses or hosts exhibiting phylogenetic uncertainty were excluded from the analysis.

## Supporting Information

Figure S1 Comparison of genetic characteristics among the novel hantavirus sequences obtained here with other known members of the genus *Hantavirus*. Panel A shows the N protein and Ns protein encoded by the S segment. Panel B shows the GPC protein. The numbers and black lines represent the potential N-linked glycosylation sites; the yellow box represents the Gn protein; the green box represents the Gc protein; the red box represents the amino acid cleavage site.
(PDF)

Figure S2 Phylogenetic trees based on the N (A, B, C), GPC (D, E, F), and partial L (G, H, I) protein sequences of hantaviruses including the viruses obtained in this study estimated using ML and Bayesian methods. Numbers (>70%/>0.7/>0.7) above or below branches indicate posterior node probabilities or bootstrap values. Scale bar represents number of amino acid substitutions per site.
(PDF)

Figure S3 MCC (A) and the Bayesian (B) phylogenetic trees of partial L segment sequences of hantaviruses including the viruses obtained in this study. Numbers (>0.7/>0.7) above or below branches indicate posterior node probabilities or bootstrap values. Scale bar represents the number of nucleotide substitutions per site.
(PDF)

Figure S4 Tanglegram constructed using TreeMap2.0b illustrating the phylogenies of hantaviruses and their bat, insectivore, and rodent hosts. (A) The host tree on the left was based on mt-*cyt b* gene sequences, and the hantavirus tree on the right was based on the coding sequences of M segment. (B) The host tree on the left was based on cytochrome b gene sequences, and the hantavirus tree on the right was based on the coding sequences of S+M segment. Numbers (>0.7) above or below branches indicate posterior node probabilities.
(PDF)

Table S1 Hantavirus sequences obtained in this study and from GenBank.
(DOC)

Table S2 Percentage similarities of S and M segments among the new hantaviruses identified here and other hantaviruses.
(DOC)

Table S3 Percentage similarities of partial L segments among the new hantaviruses identified here and other hantaviruses.
(DOC)

Table S4 Mitochondrial *cyt b* sequences of mammalian hosts obtained in this study and from GenBank.
(DOC)

Table S5 Results of the host phylogenetic reconciliation analysis.
(DOC)

Table S6 Specific primers used in this study.
(DOC)
