# Supplementary figures and images for: Phylogeny and Origins of Hantaviruses Harbored by Bats, Insectivores, and Rodents
Source: PLoS Pathog. 2013 Feb 7;9(2):e1003159. doi: 10.1371/journal.ppat.1003159 (PMC3567184; doi:10.1371/journal.ppat.1003159)

**A**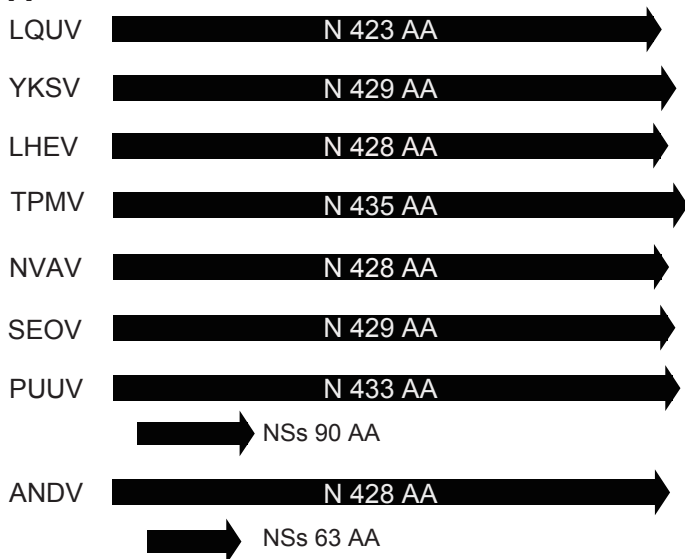**B**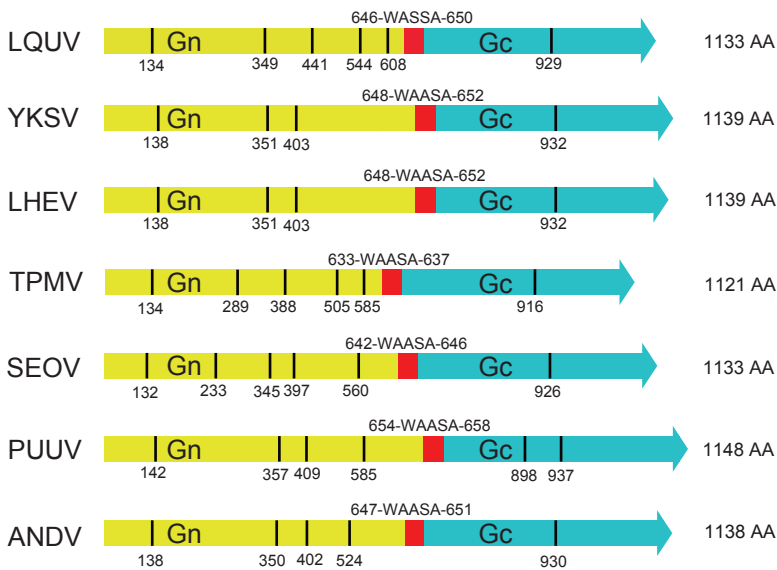

Supplement: Figure S1 — Comparison of genetic characteristics among the novel hantavirus sequences obtained here with other known members of the genus Hantavirus. Panel A shows the N protein and Ns protein encoded by the S segment. Panel B shows the GPC protein. The numbers and black lines represent the potential N-linked glycosylation sites; the yellow box represents the Gn protein; the green box represents the Gc protein; the red box represents the amino acid cleavage site. (PDF) [file ppat.1003159.s001.pdf]

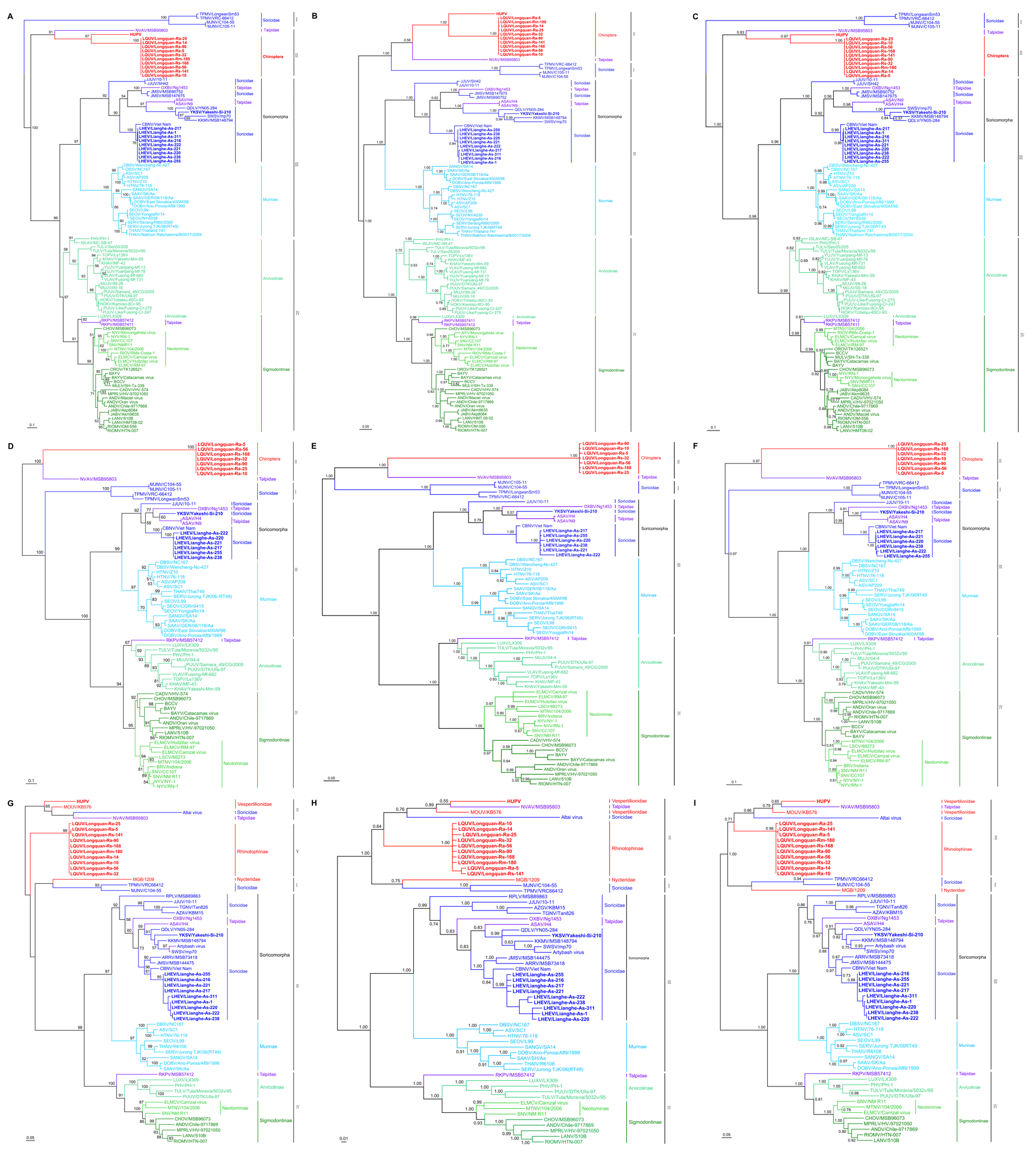

Supplement: Figure S2 — Phylogenetic trees based on the N (A, B, C), GPC (D, E, F), and partial L (G, H, I) protein sequences of hantaviruses including the viruses obtained in this study estimated using ML and Bayesian methods. Numbers (>70%/>0.7/>0.7) above or below branches indicate posterior node probabilities or bootstrap values. Scale bar represents number of amino acid substitutions per site. (PDF) [file ppat.1003159.s002.pdf]

A

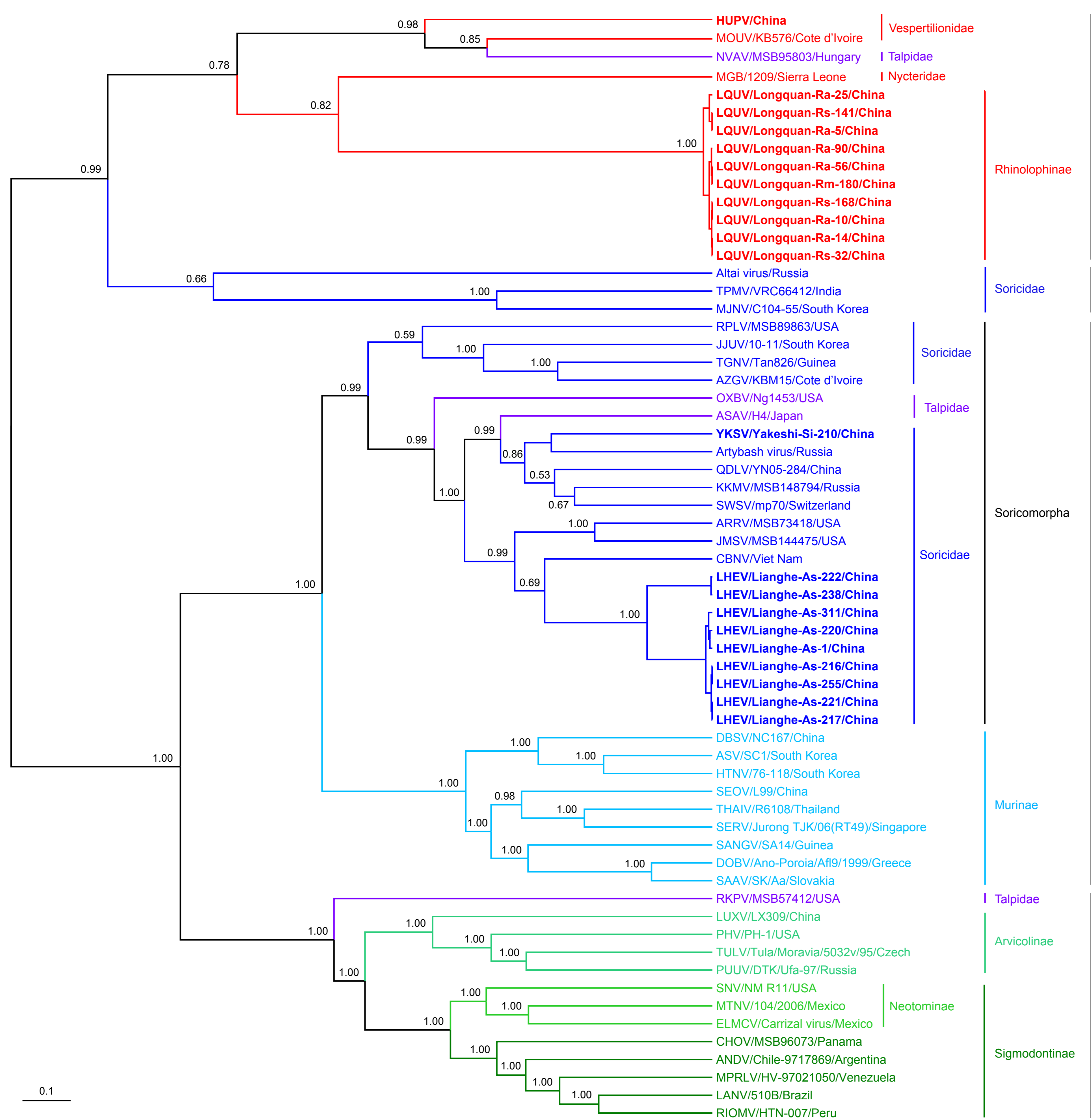

B

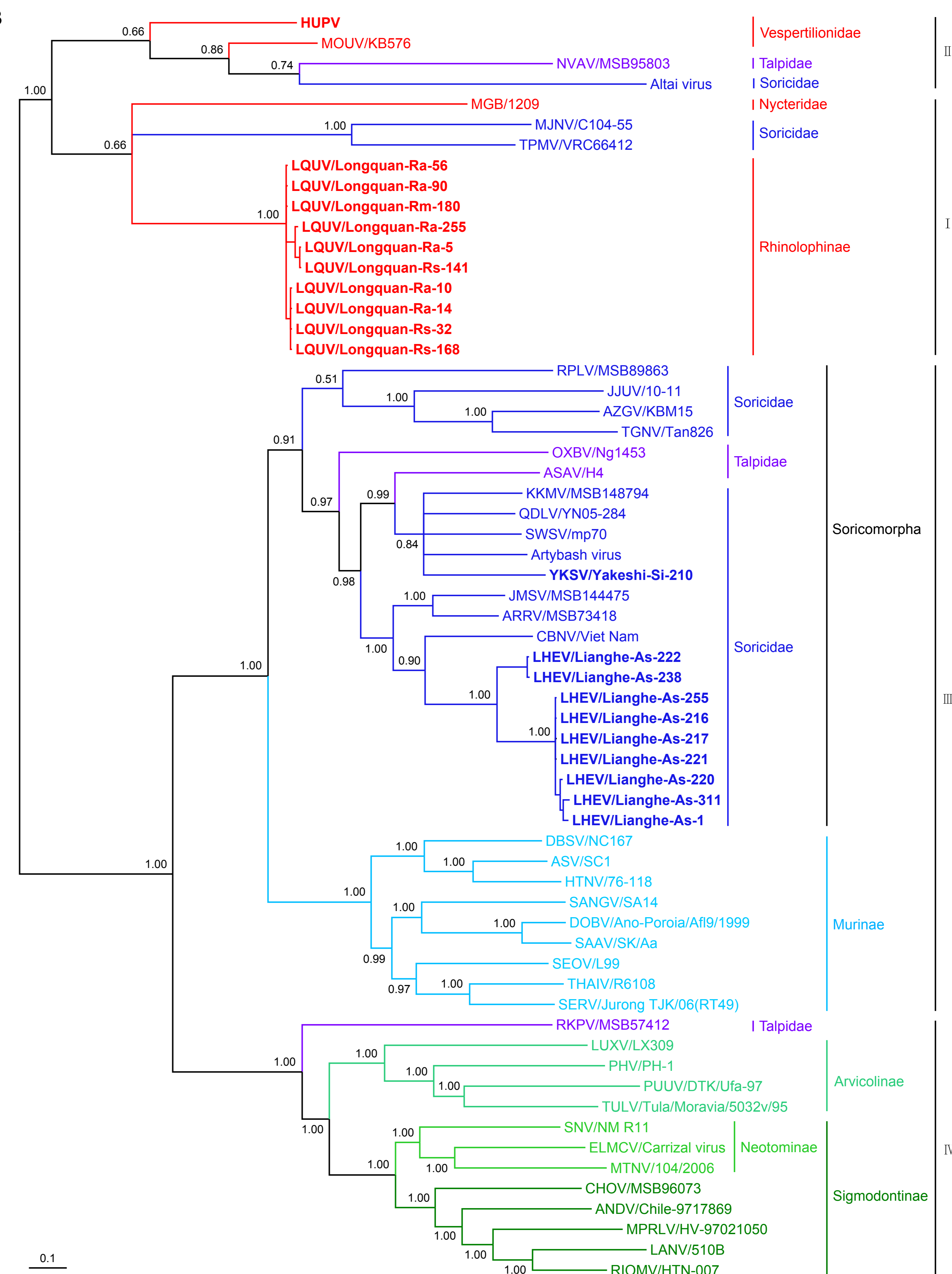

Supplement: Figure S3 — MCC (A) and the Bayesian (B) phylogenetic trees of partial L segment sequences of hantaviruses including the viruses obtained in this study. Numbers (>0.7/>0.7) above or below branches indicate posterior node probabilities or bootstrap values. Scale bar represents the number of nucleotide substitutions per site. (PDF) [file ppat.1003159.s003.pdf]

A

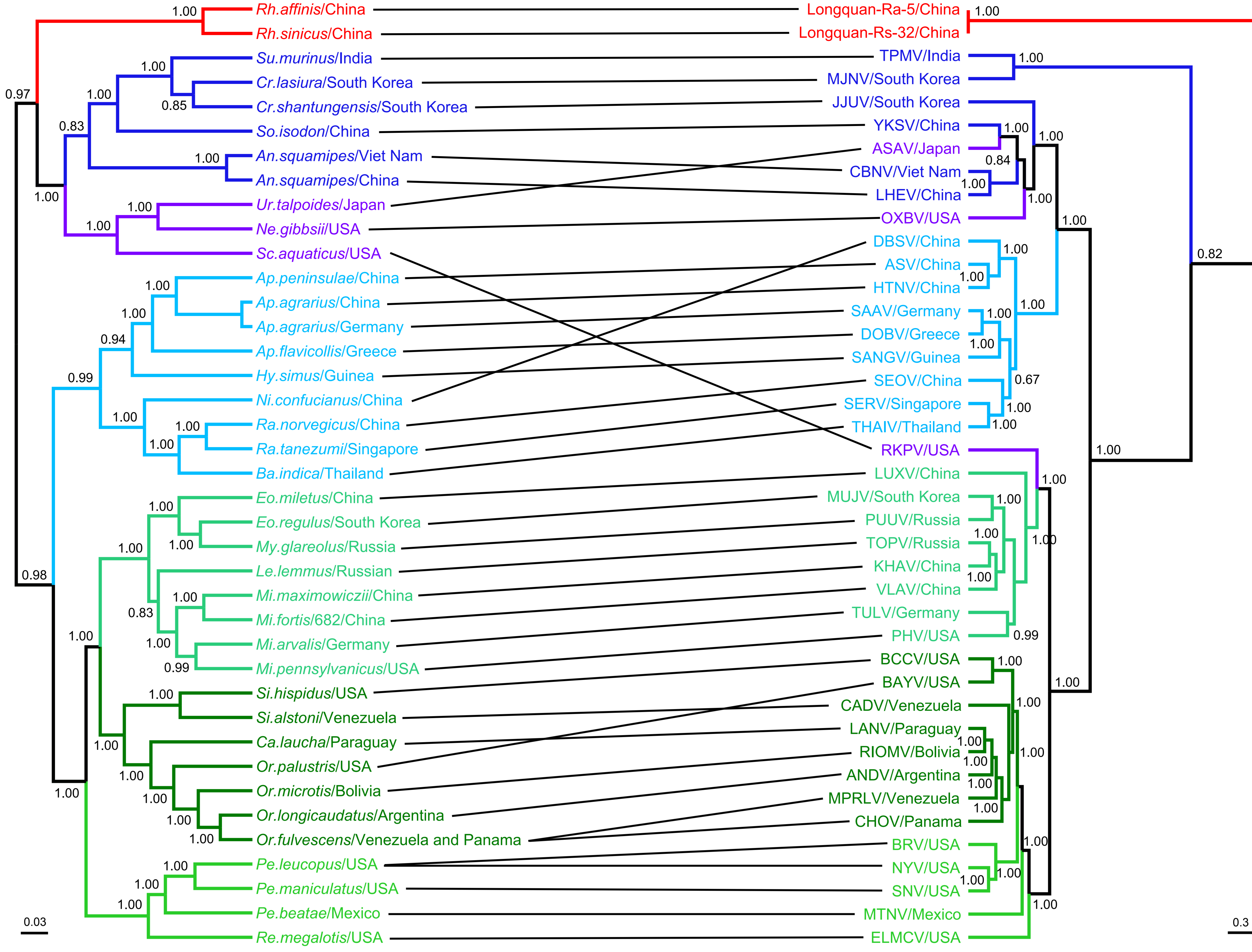

B

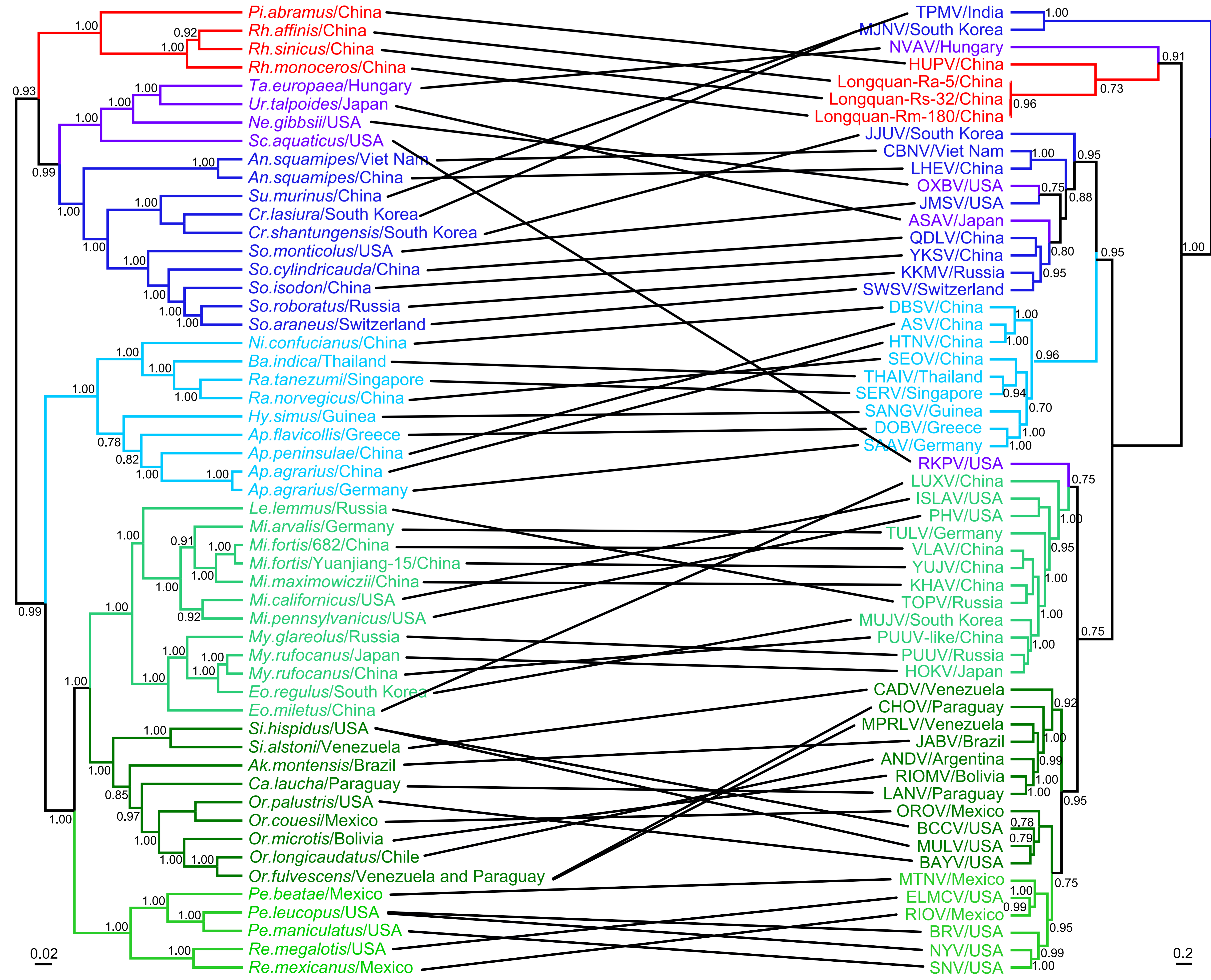

Supplement: Figure S4 — Tanglegram constructed using TreeMap2.0b illustrating the phylogenies of hantaviruses and their bat, insectivore, and rodent hosts. (A) The host tree on the left was based on mt-cyt b gene sequences, and the hantavirus tree on the right was based on the coding sequences of M segment. (B) The host tree on the left was based on cytochrome b gene sequences, and the hantavirus tree on the right was based on the coding sequences of S+M segment. Numbers (>0.7) above or below branches indicate posterior node probabilities. (PDF) [file ppat.1003159.s004.pdf]
