# Supplementary material for: Phylogeny and Origins of Hantaviruses Harbored by Bats, Insectivores, and Rodents
Source: PLoS Pathog. 2013 Feb 7;9(2):e1003159. doi: 10.1371/journal.ppat.1003159 (PMC3567184; doi:10.1371/journal.ppat.1003159)
Supplement: Table S1 — Hantavirus sequences obtained in this study and from GenBank. (DOC) [file ppat.1003159.s005.doc]

| Strain | GenBank | | | Note |
| --- | --- | --- | --- | --- |
|  | S | M | L |  |
| **Bats-related HV** | | | | |
| LQUV/Longquan-Ra-5 | JX465416 | - | JX465382 | This study |
| LQUV/Longquan-Ra-10 | JX465413 | JX465398 | JX465379 | This study |
| LQUV/Longquan-Ra-14 | JX465414 | - | JX465380 | This study |
| LQUV/Longquan-Ra-25 | JX465415 | JX465397 | JX465381 | This study |
| LQUV/Longquan-Rs-32 | JX465422 | JX465402 | JX465388 | This study |
| LQUV/Longquan-Ra-56 | JX465417 | JX465399 | JX465383 | This study |
| LQUV/Longquan-Ra-90 | JX465418 | JX465400 | JX465384 | This study |
| LQUV/Longquan-Rs-141 | JX465420 | - | JX465386 | This study |
| LQUV/Longquan-Rs-168 | JX465421 | JX465401 | JX465387 | This study |
| LQUV/Longquan-Rm-180 | JX465419 | - | JX465385 | This study |
| HUPV/Huangpi-Pa-1 | JX473273 | - | JX465369 | This study |
| MOUV/KB576 | - | - | JQ287716 | GenBank |
| MGB/1209 | - | - | JN037851 | GenBank |
| **Insectivore-related HV** | | | | |
| TPMV/LongwanSm53 | JF784172 | JF784178 | - | GenBank |
| TPMV/VRC66412 | AY526097 | NC_010708 | NC_010707 | GenBank |
| MJNV/C104-55 | EF641805 | EF641799 | EF641807 | GenBank |
| MJNV/C105-11 | EF641804 | EF641806 | - | GenBank |
| NVAV/MSB95803 | FJ539168 | HQ840957(partial) | FJ593498 | GenBank |
| QDLV/YN05-284 | GU566023 | - | GU566021 | GenBank |
| JJUV/SH42 | HQ663933 | - | - | GenBank |
| JJUV/10-11 | HQ834695 | HQ834696 | HQ834697 | GenBank |
| OXBV/Ng1453 | FJ539166 | FJ539167 | FJ593497 | GenBank |
| JMSV/MSB147675 | FJ686863 | - | - | GenBank |
| JMSV/MSB90752 | FJ686859 | - | - | GenBank |
| JMSV/MSB144475 | - | - | FJ593501 | GenBank |
| ASAV/H4 | EU929070 | EU929073 | EU929076 | GenBank |
| ASAV/N9 | EU929071 | EU929074 | - | GenBank |
| SWSV/mp70 | EF636024 | - | EF636026 | GenBank |
| KKMV/MSB148794 | GQ306148 | - | GQ306150 | GenBank |
| RKPV/MSB57411 | HM015218 | - | - | GenBank |
| RKPV/MSB57412 | HM015223 | HM015219 | HM015221 | GenBank |
| CBNV/Viet Nam | EF543524 | EF543526 | EF543525 | GenBank |
| LHEV/Lianghe-As-1 | JX465404 | - | JX465370 | This study |
| LHEV/Lianghe-As-216 | JX465405 | - | JX465371 | This study |
| LHEV/Lianghe-As-217 | JX465406 | JX465390 | JX465372 | This study |
| LHEV/Lianghe-As-220 | JX465407 | JX465391 | JX465373 | This study |
| LHEV/Lianghe-As-221 | JX465408 | JX465392 | JX465374 | This study |

Table S1. Hantavirus sequences obtained in this study and from GenBank

| LHEV/Lianghe-As-222 | JX465409 | JX465393 | JX465375 | This study |
| --- | --- | --- | --- | --- |
| LHEV/Lianghe-As-238 | JX465410 | JX465394 | JX465376 | This study |
| LHEV/Lianghe-As-255 | JX465411 | JX465395 | JX465377 | This study |
| LHEV/Lianghe-As-311 | JX465412 | - | JX465378 | This study |
| YKSV/Yakeshi-Si-210 | JX465423 | JX465403 | JX465389 | This study |
| TGNV/Tan826 | - | - | EF050454 | GenBank |
| AZGV/KBM15 | - | - | JF272628 | GenBank |
| ARRV/MSB73418 | - | - | EF619961 | GenBank |
| RPLV/MSB89863 | - | - | EF540771 | GenBank |
| Artybash virus | - | - | EU424339 | GenBank |
| Altai virus |  |  | EU424341 | GenBank |
| **Mur-related HV** | | | | |
| HTNV/76-118 | M14626 | Y00386 | X55901 | GenBank |
| HTNV/Z10 | EF533944 | AF143675 | - | GenBank |
| ASV/AP209 | AB620028 | AB620029 | - | GenBank |
| ASV/SC1 | AY675349 | AY675353 | DQ056292 | GenBank |
| DBSV/NC167 | AB027523 | AB027115 | DQ989237 | GenBank |
| DBSV/Wencheng-Nc-427 | GF796017 | GF796031 | - | GenBank |
| DOBV/East Slovakia/400Af/98 | AY168576 | AY168577 | - | GenBank |
| DOBV/Ano-Poroia/Afl9/1999 | AJ410615 | AJ410616 | AJ410617 | GenBank |
| SAAV/GER/08/118/Aa | GQ205407 | GQ205412 | - | GenBank |
| SAAV/SK/Aa | AY961615 | AY961616 | GU904039 | GenBank |
| SANGV/SA14 | JQ082300 | JQ082301 | JQ082302 | GenBank |
| SEOV/L99 | AF288299 | AF288298 | AF288297 | GenBank |
| SEOV/NYA039 | EF210131 | - | - | GenBank |
| SEOV/YongjiaRn14 | GU592947 | GU592827 | - | GenBank |
| SEOV/CGRn9415 | - | EF990916 | - | GenBank |
| THAIV/Thailand 741 | AB186420 | - | - | GenBank |
| THAIV/Nakhon Ratchasima/Bi0017/2004 | AM397664 | - | - | GenBank |
| THAIV/Thai749 | - | L08756 |  | GenBank |
| THAIV/R6108 | - | - | JN116259 | GenBank |
| Serang/Rt60/2000 | AM998808 | - | AM998806 | GenBank |
| Jurong TJK/06(RT49) | GQ274940 | GQ274938 | GQ274936 | GenBank |
| **Arv-related HV** | | | | |
| LXV/LX309 | HM756286 | HM756287 | HQ404253 | GenBank |
| HOKV/Tobetsu-60Cr-93 | AB010731 | - | - | GenBank |
| HOKV/Kamiiso-8Cr-95 | AB010730 | - | - | GenBank |
| PUUV/DTK/Ufa-97 | AB297665 | AB297662 | AB297667 | GenBank |
| PUUV/Samara_49/CG/2005 | AB433842 | AB433850 | - | GenBank |
| PUUV-Like/Fusong-Cr-247 | EF442087 | - | - | GenBank |

| PUUV-Like/Fusong-Cr-275 | EF442091 | - | - | GenBank |
| --- | --- | --- | --- | --- |
| MUJV/04-4 | - | EF198413 | - | GenBank |
| MUJV/00-18 | DQ138128 | - | - | GenBank |
| MUJV/99-28 | DQ138142 | - | - | GenBank |
| KHAV/MF-43 | U35255 | AJ011648 | AJ011650 | GenBank |
| KHAV/Yakeshi-Mm-59 | EU072483 | EU072489 | - | GenBank |
| TOPV/Ls136V | AJ011646 | AJ011647 | AJ011649 | GenBank |
| VLAV/Fusong-Mf-682 | EU072480 | EU072488 | FJ170807 | GenBank |
| VLAV/Fusong-Mf-731 | EU072481 | - | - | GenBank |
| YUJV/Yuanjiang-Mf-13 | FJ170795 | - | - | GenBank |
| YUJV/Yuanjiang-Mf-78 | FJ170792 | - | FJ170811 | GenBank |
| TULV/Tula/Moravia/5032v/95 | Z69991 | Z69993 | AJ005637 | GenBank |
| TULV/Sen05/205 | EU439951 | - | - | GenBank |
| PHV/PH-1 | Z49098 | X55129 | EF646763 | GenBank |
| ISLAV/MC-SB-47 | U19302 | - | - | GenBank |
| **Sig-related HV** | | | | |
| BAYV | L36929 | L36930 | - | GenBank |
| BAYV/Catacamas virus | DQ256126 | DQ177347 | - | GenBank |
| BCCV | L39949 | L39950 | - | GenBank |
| MULV/SH-Tx-339 | U54575 | - | - | GenBank |
| OROV/TK126521 | EF534079 | - | - | GenBank |
| CADV/VHV-574 | DQ285566 | DQ284451 | - | GenBank |
| CHOV/MSB96073 | DQ285046 | DQ285047 | EF397003 | GenBank |
| ELMCV/Carrizal virus | AB620103 | AB620104 | AB620105 | GenBank |
| ELMCV/Huitzilac virus | AB620106 | AB620107 | - | GenBank |
| ELMCV/RM-97 | U11427 | U26828 | - | GenBank |
| RIOV/RMx-Costa-1 | U18100 | - | - | GenBank |
| MTNV/104/2006 | AB620100 | AB620101 | AB620102 | GenBank |
| NYV/Monongahela virus | U32591 | - | - | GenBank |
| NYV/RN-1 | U09488 | U36801 | - | GenBank |
| NYV/NY-1 | - | U36802 | - | GenBank |
| BRV/Indiana | - | AF030551 | - | GenBank |
| SNV/CC107 | L33683 | L33474 | - | GenBank |
| SNV/NM R11 | L37904 | L37903 | L37902 | GenBank |
| ANDV/Chile-9717869 | AF291702 | AF291703 | AF291704 | GenBank |
| ANDV/Maciel virus | AF482716 | - | - | GenBank |
| ANDV/Oran virus | AF482715 | AF028024 | - | GenBank |
| LANV/HMT 08-02 | FJ816031 | - | - | GenBank |
| LANV/510B | AF005727 | AF005728 | AF005729 | GenBank |
| RIOMV/OM-556 | U52136 | - | - | GenBank |
| RIOMV/HTN-007 | FJ532244 | FJ608550 | FJ809772 | GenBank |
| MPRLV/HV-97021050 | AY267347 | AY363179 | EU788002 | GenBank |

| JABV/Akm9635 | JN232078 | - | - | GenBank |
| --- | --- | --- | --- | --- |
| JABV/Akp8084 | JN232080 | - | - | GenBank |
| LSCV:68273 | - | AF307323 | - | GenBank |

Note: “-” sequences not available or used in this study.
