# Supplementary material for: Phylogeny and Origins of Hantaviruses Harbored by Bats, Insectivores, and Rodents
Source: PLoS Pathog. 2013 Feb 7;9(2):e1003159. doi: 10.1371/journal.ppat.1003159 (PMC3567184; doi:10.1371/journal.ppat.1003159)
Supplement: Table S2 — Percentage similarities of S and M segments among the new hantaviruses identified here and other hantaviruses. (DOC) [file ppat.1003159.s006.doc]

|  | 1 | 2 | 3 | 4 | 5 | 6 | 7 | 8 | 9 | 10 | 11 | 12 | 13 |
| --- | --- | --- | --- | --- | --- | --- | --- | --- | --- | --- | --- | --- | --- |
| S segment |  |  |  |  |  |  |  |  |  |  |  |  |  |
| 1 LQUV |  | 64.2-65.2 | 52.6-53.0 | 50.5-50.8 | 48.8-57.9 | 55.7-55.8 | 52.7-53.5 | 49.5-61.2 | 50.2-62.0 | 50.7-53.5 | 49.0-54.6 | 53.0-53.3 | 54.5-56.3 |
| 2 HUPV(partial) | 68.2-68.5 |  | 60.4-61.0 | 59.7 | 57.9-59.9 | 62.9 | 63.3 | 59.9-65.3 | 59.9-63.9 | 62.0-67.0 | 60.0-64.4 | 62.0 | 64.8-65.0 |
| 3 LHEV | 51.8-52.5 | 58.1-58.4 |  | 69.4-69.9 | 48.2-52.9 | 56.6-57.1 | 82.3-82.6 | 63.2-67.1 | 66.7-73.4 | 63.1-66.2 | 61.4-65.8 | 65.1-65.4 | 61.5-64.0 |
| 4 YKSV | 52.7 | 54.3 | 68.9-69.4 |  | 46.6-51.4 | 53.5 | 66.7 | 51.3-65.7 | 64.7-76.3 | 59.3-65.4 | 53.7-64.3 | 63.8 | 64.4 |
| 5 Group I | 45.2-47.3 | 49.8-52.4 | 46.7-48.8 | 44.8-45.9 |  | 49.5-57.0 | 49.8-51.9 | 47.4-54.3 | 46.8-60.6 | 46.3-52.6 | 48.5-58.6 | 47.9-50.1 | 47.9-50.2 |
| 6 NVAV | 54.8-55.3 | 58.8 | 49.8-50.0 | 49.5 | 45.3-46.0 |  | 58.3 | 51.6-59.8 | 49.5-58.3 | 51.4-60.5 | 51.3-61.4 | 51.6 | 51.4 |
| 7 CBNV | 53.0-53.2 | 58.8 | 94.9-95.6 | 68.7 | 47.7-48.4 | 50.2 |  | 63.1-66.5 | 65.8-71.8 | 63.0-66.0 | 53.6-64.4 | 64.6 | 64.3 |
| 8 Murinae | 51.1-55.6 | 55.1-58.8 | 61.7-64.0 | 59.4-63.2 | 45.0-48.0 | 48.8-51.2 | 62.1-65.0 |  | 52.2-68.1 | 63.6-67.4 | 63.2-67.9 | 65.2-67.4 | 64.2-66.0 |
| 9 Soricomorpha | 50.1-52.2 | 54.7-60.3 | 66.8-79.2 | 63.1-86.0 | 43.1-47.6 | 48.4-50.7 | 67.3-77.6 | 57.8-65.0 |  | 53.1-66.4 | 52.6-66.0 | 53.7-64.2 | 53.0-65.1 |
| 10 Arvicolinae | 51.8-54.4 | 54.3-58.8 | 59.1-63.3 | 58.0-61.8 | 44.3-46.9 | 50.0-53.5 | 58.2-63.1 | 60.6-64.1 | 56.8-61.1 |  | 68.4-73.1 | 70.4-74.0 | 68.5-71.7 |
| 11 Sigmodontinae | 51.8-54.6 | 56.6-59.6 | 58.6-62.1 | 57.9-60.0 | 43.9-48.6 | 50.2-52.8 | 58.9-61.9 | 59.6-66.4 | 54.9-61.0 | 70.1-77.3 |  | 71.1-75.2 | 69.3-71.7 |
| 12 LXUV | 53.4-53.9 | 59.6 | 60.7-61.2 | 59.9 | 44.7-45.3 | 52.6 | 60.3 | 61.5-64.3 | 56.3-60.0 | 73.2-79.5 | 74.5-80.4 |  | 72.6-72.7 |
| 13 RKPV | 54.1-54.4 | 58.8 | 61.0-61.7 | 61.0 | 46.0-47.0 | 52.3 | 61.4 | 61.2-64.3 | 57.5-61.0 | 70.8-77.8 | 76.2-79.2 | 80.8 |  |
|  |  |  |  |  |  |  |  |  |  |  |  |  |  |
| M segment |  |  |  |  |  |  |  |  |  |  |  |  |  |
| 1 LQUV |  |  | 54.5-55.4 | 41.1-41.4 | 41.5-43.9 | 56.8-58.2 | 54.8-55.1 | 40.3-53.9 | 39.8-53.3 | 39.0-56.8 | 38.9-57.1 | 54.5-54.7 | 58.3-58.5 |
| 2 HUPV(partial) |  |  |  |  |  |  |  |  |  |  |  |  |  |
| 3 LHEV | 38.0-39.4 |  |  | 69.6-70.6 | 41.8-43.5 | 54.8-56.2 | 79.0-80.2 | 58.6-64.9 | 65.7-69.7 | 55.5-61.4 | 55.1-61.7 | 59.5-60.3 | 58.7-59.5 |
| 4 YKSV | 39.5 |  | 71.5-73.7 |  | 40.6-43.6 | 52.8 | 70.9 | 62.6-65.0 | 66.2-73.0 | 58.4-61.0 | 57.8-61.3 | 59.7 | 55.1 |
| 5 Group I | 37.7-38.8 |  | 39.6-42.4 | 41.9-42.9 |  | 51.0-57.0 | 40.8-42.9 | 40.3-43.1 | 40.6-42.3 | 39.2-42.7 | 39.9-43.7 | 41.2-44.2 | 40.8-42.7 |
| 6 NVAV | 41.1-41.5 |  | 41.1-43.2 | 42.8 | 43.6-44.1 |  | 57.3 | 51.7-57.3 | 51.0-52.5 | 51.8-57.9 | 48.9-57.7 | 56.9 | 55.9 |
| 7 CBNV | 39.5-39.7 |  | 89.5-92.7 | 74.2 | 41.3-42.4 | 43.2 |  | 62.1-64.9 | 66.3-68.7 | 55.3-61.7 | 55.1-61.8 | 60.9 | 59.2 |
| 8 Murinae | 40.5-41.6 |  | 57.1-61.5 | 58.7-61.1 | 41.0-43.4 | 42.4-46.2 | 59.5-61.9 |  | 61.6-64.3 | 55.6-61.9 | 55.1-62.7 | 59.9-61.7 | 55.8-60.9 |
| 9 Soricomorpha | 38.6-39.7 |  | 63.2-70.9 | 66.1-77.0 | 40.3-42.8 | 40.3-43.6 | 64.1-71.0 | 55.3-60.2 |  | 54.9-61.6 | 55.1-61.2 | 58.6-60.3 | 56.1-60.5 |
| 10 Arvicolinae | 40.5-41.6 |  | 49.5-52.9 | 51.1-52.6 | 41.2-43.3 | 42.4-46.2 | 51.6-52.8 | 51.8-55.5 | 50.5-53.8 |  | 64.0-68.0 | 69.1-70.7 | 63.1-64.6 |
| 11 Sigmodontinae | 38.9-40.9 |  | 51.1-54.9 | 51.8-53.4 | 41.5-44.2 | 41.5-45.8 | 53.5-55.7 | 52.5-56.4 | 51.1-54.5 | 64.1-69.2 |  | 65.2-67.7 | 61.3-64.8 |
| 12 LXUV | 40.2-40.4 |  | 49.9-51.6 | 33.9 | 41.8-42.4 | 44.5 | 51.5 | 53.6-55.2 | 50.4-52.4 | 70.9-73.7 | 63.8-67.4 |  | 65.3 |
| 13 RKPV | 41.1-41.3 |  | 50.2-51.9 | 50.7 | 41.7-42.4 | 42.8 | 51.8 | 52.2-53.7 | 51.0-51.8 | 61.4-62.9 | 60.8-62.9 | 62.4 |  |

Table S2. Percentage similarities of S and M segments among the new hantaviruses identified here and other hantaviruses

Note: Above diagonal are nucleotide similarities; below diagonal are amino acid similarities.

The S segment of LQUV is partial while its M segment is unavailable.
