# Supplementary material for: Phylogeny and Origins of Hantaviruses Harbored by Bats, Insectivores, and Rodents
Source: PLoS Pathog. 2013 Feb 7;9(2):e1003159. doi: 10.1371/journal.ppat.1003159 (PMC3567184; doi:10.1371/journal.ppat.1003159)
Supplement: Table S3 — Percentage similarities of partial L segments among the new hantaviruses identified here and other hantaviruses. (DOC) [file ppat.1003159.s007.doc]

|  | 1 | 2 | 3 | 4 | 5 | 6 | 7 | 8 | 9 | 10 | 11 | 12 | 13 | 14 | 15 |
| --- | --- | --- | --- | --- | --- | --- | --- | --- | --- | --- | --- | --- | --- | --- | --- |
| 1 LQUV |  | 64.3-65.0 | 59.3-68.9 | 68.6-69.5 | 57.7-61.7 | 69.4-70.7 | 67.6-74.4 | 69.8-71.0 | 65.1-67.6 | 61.3-68.9 | 64.5-73.0 | 66.7-70.8 | 64.2-70.8 | 67.6-67.9 | 68.9-69.5 |
| 2 HUPV | 72.3-74.5 |  | 60.4-63.1 | 62.9 | 60.8 | 68.6-71.0 | 67.1 | 70.3 | 65.0 | 61.1-65.4 | 63.6-67.5 | 60.1-63.6 | 53.4-63.3 | 61.8 | 64.3 |
| 3 LHEV | 61.8-66.0 | 58.5-59.6 |  | 76.5-78.0 | 52.3-56.0 | 64.2-67.9 | 59.7-65.7 | 58.8-66.7 | 74.3-79.2 | 70.4-74.8 | 65.7-79.6 | 64.8-70.1 | 58.6-70.4 | 67.9-70.4 | 67.9-69.8 |
| 4 YKSV | 63.2 | 55.3 | 87.6-88.7 |  | 60.0 | 64.2-65.1 | 66.7-68.9 | 66.7 | 74.2 | 68.9-73.3 | 61.9-80.2 | 63.2-67.3 | 62.9-68.9 | 71.1 | 69.5 |
| 5 Altai virus | 65.0-66.0 | 64.9 | 62.0-62.9 | 59.0 |  | 58.7-60.3 | 54.7-61.3 | 62.3 | 58.0 | 52.7-62.0 | 54.7-63.0 | 51.3-61.7 | 41.7-58.7 | 56.0 | 53.7 |
| 6 bat-related virus | 69.4-77.8 | 69.1-81.9 | 60.7-64.2 | 62.3 | 65.0-66.0 |  | 68.2-71.9 | 67.0-71.6 | 64.5-65.1 | 56.6-70.1 | 59.4-70.1 | 63.8-67.3 | 55.3-67.0 | 63.5-64.2 | 66.4-67.0 |
| 7 Group I | 70.4-75.0 | 72.3-74.5 | 58.4-62.3 | 62.3 | 62.0-66.0 | 69.4-75.0 |  | 68.5-69.1 | 62.6-67.6 | 60.1-67.8 | 61.6-69.5 | 62.6-66.4 | 63.2-68.2 | 63.2-67.0 | 64.2-65.4 |
| 8 NVAV | 70.4-71.3 | 75.5 | 62.9-64.2 | 63.2 | 67.0 | 67.6-78.7 | 71.3-73.1 |  | 64.5 | 60.7-66.0 | 60.1-66.7 | 61.3-63.8 | 56.6-65.1 | 64.2 | 67.9 |
| 9 CBNV | 66.0-67.0 | 57.4 | 93.2-94.3 | 85.8 | 63.0 | 60.4-63.2 | 61.3 | 63.2 |  | 69.2-74.2 | 65.4-77.9 | 66.4-69.5 | 64.2-68.2 | 64.2 | 64.5 |
| 10 Murinae | 62.3-68.6 | 58.5-66.0 | 75.5-80.2 | 73.6-75.5 | 54.0-58.0 | 54.7-69.8 | 60.4-67.9 | 58.5-66.0 | 76.4-80.2 |  | 64.5-74.5 | 63.5-70.4 | 58.2-71.4 | 63.2-67.9 | 61.6-71.0 |
| 11 Soricomorpha | 62.7-69.8 | 57.4-63.8 | 66.3-91.5 | 70.8-89.6 | 54.0-62.0 | 59.4-69.8 | 59.8-69.8 | 57.5-67.0 | 71.7-89.2 | 65.1-80.2 |  | 64.5-72.3 | 58.2-70.8 | 65.1-72.3 | 62.9-72.0 |
| 12 Arvicolinae | 63.2-66.0 | 54.3-57.4 | 62.9-67.9 | 66.6-67.9 | 54.0-57.0 | 60.4-65.1 | 60.4-63.2 | 58.5-63.2 | 66.0-67.9 | 62.3-68.9 | 61.8-70.8 |  | 68.6-74.5 | 69.8-74.5 | 701.-72.6 |
| 13 Sigmodontinae | 61.3-67.0 | 54.3-60.6 | 58.4-72.6 | 61.3-70.8 | 53.0-56.0 | 56.6-66.0 | 60.4-67.9 | 59.4-63.2 | 62.3-71.7 | 58.5-70.8 | 58.8-72.6 | 75.5-80.2 |  | 69.5-72.3 | 68.6-74.5 |
| 14 LXUV | 60.4-61.3 | 55.3 | 61.8-66.0 | 65.1 | 53.0 | 57.5-61.3 | 60.4-63.2 | 57.5 | 63.2 | 61.3-67.0 | 63.2-67.9 | 77.4-83.0 | 71.7-77.4 |  | 70.8 |
| 15 RKPV | 65.1-66.0 | 60.6 | 59.6-63.2 | 63.2 | 54.0 | 60.4-67.9 | 64.2-66.0 | 63.2 | 62.3 | 60.4-69.8 | 59.8-67.9 | 74.5-79.2 | 68.9-74.5 | 72.6 |  |

Table S3. Percentage similarities of partial L segments among the new hantaviruses identified here and other hantaviruses
