# Supplementary material for: Phylogeny and Origins of Hantaviruses Harbored by Bats, Insectivores, and Rodents
Source: PLoS Pathog. 2013 Feb 7;9(2):e1003159. doi: 10.1371/journal.ppat.1003159 (PMC3567184; doi:10.1371/journal.ppat.1003159)
Supplement: Table S4 — Mitochondrial cyt b sequences of mammalian hosts obtained in this study and from GenBank. (DOC) [file ppat.1003159.s008.doc]

Table S4. Mitochondrial *cyt b* sequences of mammalian hosts obtained in this study and from GenBank

|  | Species | GenBank | Note |
| --- | --- | --- | --- |
| Bat | | | |
|  | *Nycteris hispida* | HQ693723 | GenBank |
|  | *Miniopterus schreibersii* | AF376830 | GenBank |
|  | *Neoromicia nanus* | EU797428 | GenBank |
|  | *Pipistrellus abramus* | NC_005436 | GenBank |
|  | *Ia io* | DQ302094 | GenBank |
|  | *Murina leucogaster* | AB085733 | GenBank |
|  | *Myotis altarium* | EF553530 | GenBank |
|  | *Myotis chinensis* | AB106588 | GenBank |
|  | *Hipposideros armiger* | DQ297585 | GenBank |
|  | *Rhinolophus ferrumequinum* | EU436673 | GenBank |
|  | *Rhinolophus affinis* | EF544419 | GenBank |
|  | *Rhinolophus sinicus* | EU434941 | GenBank |
|  | *Rhinolophus pearsonii* | GQ257345 | GenBank |
|  | *Rhinolophus macrotis* | EU075213 | GenBank |
|  | *Rhinolophus pusillus* | EF217389 | GenBank |
|  | *Rhinolophus monoceros* | DQ297580 | GenBank |
|  | JingmenMs6 | JX465353 | This study |
|  | HuangpiPa1 | JX465352 | This study |
|  | YichangIi81 | JX465365 | This study |
|  | YichangMl1 | JX465367 | This study |
|  | YichangMa49 | JX465366 | This study |
|  | ZaoxiMc1 | JX465368 | This study |
|  | WenchengHa7 | JX465363 | This study |
|  | JingmenRf7 | JX465354 | This study |
|  | LongquanRa5 | JX465358 | This study |
|  | LongquanRs32 | JX465362 | This study |
|  | LongquanRp77 | JX465360 | This study |
|  | LongquanRm180 | JX465359 | This study |
|  | JingmenRm28 | JX465355 | This study |
|  | JingmenRp14 | JX465356 | This study |
|  | LongquanRp94 | JX465361 | This study |
| Insectivore | | | |
|  | *Scalopus aquaticus* | HM461915 | GenBank |
|  | *Neurotrichus gibbsii* | AB076821 | GenBank |
|  | *Talpa europaea* | FJ715340 | GenBank |
|  | *Urotrichus talpoides* | AB099483 | GenBank |
|  | *Anourosorex squamipes* | JX465357 | This study |
|  | *Anourosorex squamipes*/Viet Nam | EF543528 | GenBank |

|  | *Crocidura lasiura* | AB077072 | GenBank |
| --- | --- | --- | --- |
|  | *Crocidura shantungensis* | HQ709236 | GenBank |
|  | *Suncus murinus* | JF784169 | GenBank |
|  | *Sorex cinereus* | JN889696 | GenBank |
|  | *Sorex monticolus* | AB100273 | GenBank |
|  | *Sorex cylindricauda* | GU566020 | GenBank |
|  | *Sorex araneus* | GU564731 | GenBank |
|  | *Sorex roboratus* | GQ374411 | GenBank |
|  | *Sorex isodon* | JX465364 | This study |
|  | *Scaptonyx fusicaudus* | AB106229 | GenBank |
|  | *Crocidura obscurior* | JF276229 | GenBank |
|  | *Crocidura theresae* | DQ305278 | GenBank |
|  | *Blarina brevicauda* | AF533613 | GenBank |
|  | *Blarina carolinensis* | AF395459 | GenBank |
|  | *Sorex trowbridgii* | AY014956 | GenBank |
|  | *Sorex vagrans* | AF154551 | GenBank |
|  | *Sorex palustris* | AF238033 | GenBank |
|  | *Sorex caecutiens* | AB028547 | GenBank |
| Rodent | | | |
|  | *Niviventer confucianus* | JF796024 | GenBank |
|  | *Bandicota indica* | HM217425 | GenBank |
|  | *Rattus norvegicus* | GU592958 | GenBank |
|  | *Rattus tanezumi* | GQ274948 | GenBank |
|  | *Apodemus flavicollis* | JF819967 | GenBank |
|  | *Apodemus peninsulae* | AY388999 | GenBank |
|  | *Apodemus agrarius*/Europe | JF318967 | GenBank |
|  | *Apodemus agrarius*/China | AB096809 | GenBank |
|  | *Mastomys natalensis* | HM130519 | GenBank |
|  | *Zelotomys hildegardeae* | EU349791 | GenBank |
|  | *Praomys rostratus* | JQ735857 | GenBank |
|  | *Hylomyscus simus* | DQ212188 | GenBank |
|  | *Hylomyscus pamfi* | HM013792 | GenBank |
|  | *Reithrodontomys megalotis* | HQ269731 | GenBank |
|  | *Reithrodontomys mexicanus* | AY859452 | GenBank |
|  | *Peromyscus beatae* | AB618713 | GenBank |
|  | *Peromyscus boylii* | DQ000478 | GenBank |
|  | *Peromyscus leucopus* | DQ000483 | GenBank |
|  | *Peromyscus maniculatus* | FJ415095 | GenBank |
|  | *Sigmodon alstoni* | AF293397 | GenBank |
|  | *Sigmodon hispidus* | AF155420 | GenBank |
|  | *Akodon montensis* | EU251018 | GenBank |

|  | *Calomys laucha* | AF385593 | GenBank |
| --- | --- | --- | --- |
|  | *Oryzomys palustris* | EU074639 | GenBank |
|  | *Oryzomys couesi* | EU074667 | GenBank |
|  | *Oligoryzomys microtis* | EU258549 | GenBank |
|  | *Oligoryzomys longicaudatus* | GU393998 | GenBank |
|  | *Oligoryzomys fulvescens* | GU393997 | GenBank |
|  | *Eothenomys miletus* | AY426685 | GenBank |
|  | *Myodes glareolus* | EU523552 | GenBank |
|  | *Eothenomys regulus* | JN629046 | GenBank |
|  | *Myodes rufocanus* | EF442098 | GenBank |
|  | *Myodes rufocanus* | AB675439 | GenBank |
|  | *Lemmus lemmus* | AY219145 | GenBank |
|  | *Microtus arvalis* | GU190663 | GenBank |
|  | *Microtus pennsylvanicus* | AF119279 | GenBank |
|  | *Microtus californicus* | EF506194 | GenBank |
|  | *Microtus maximowiczii* | FJ986312 | GenBank |
|  | *Microtus fortis*/Yuanjiang-15 | Eu870636 | GenBank |
|  | *Microtus fortis*/682 | EU126809 | GenBank |
|  | *Bolomys lasiurus* | EF622509 | GenBank |
|  | *Zygodontomys brevicauda* | GU126549 | GenBank |
| Outgroup | | | |
|  | *Ornithorhynchus anatinus* | HQ379928 | GenBank |
