# Supplementary material for: Phylogeny and Origins of Hantaviruses Harbored by Bats, Insectivores, and Rodents
Source: PLoS Pathog. 2013 Feb 7;9(2):e1003159. doi: 10.1371/journal.ppat.1003159 (PMC3567184; doi:10.1371/journal.ppat.1003159)
Supplement: Table S5 — Results of the host phylogenetic reconciliation analysis. (DOC) [file ppat.1003159.s009.doc]

| Segment | Topology Analyzed | Number of POpt | Range of CEs in POpt | Range of NCEs in POpt | Significance of CEs (*P*) | Significance of NCEs (*P*) |
| --- | --- | --- | --- | --- | --- | --- |
| S | Arvicolinae only | 1 | 12 | 12 | 0.006±0.002 | 0.005±0.002 |
|  | Murinae only | 2 | 4 | 18 | 0.6±0.015 | 0.864±0.010 |
|  | Signodontinae only | 15 | 10 | 29 | 0.041±0.006 | 0.01±0.003 |
|  | Soricomorpha and Chiroptera only | 15 | 8 | 36 | 0.148±0.011 | 0.153±0.011 |
|  | Composite | 2 | 8 | 24 | 0.098±0.009 | 0.1±0.009 |
| M | Arvicolinae only | 1 | 8 | 6 | 0.025±0.004 | 0.021±0.004 |
|  | Murinae only | 1 | 4 | 18 | 0.621±0.015 | 0.634±0.015 |
|  | Signodontinae only | 12 | 10 | 21 | 0.025±0.004 | 0.018±0.004 |
|  | Soricomorpha and Chiroptera only | 2 | 8 | 15 | 0.046±0.006 | 0.049±0.006 |
|  | Composite | 15 | 6 | 24 | 0.297±0.014 | 0.317±0.014 |
| S and M | Arvicolinae only | 1 | 14 | 9 | 0 | 0 |
|  | Murinae only | 1 | 12 | 14 | 0.004±0.001 | 0.002±0.001 |
|  | Signodontinae only | 14 | 10 | 32 | - | - |
|  | Soricomorpha and Chiroptera only | 14 | 14 | 27 | 0 | 0 |
|  | Composite | 4 | 8 | 24 | 0.086±0.008 | 0.110±0.009 |

Table S5. Results of the host phylogenetic reconciliation analysis
