# Supplementary material for: Phylogeny and Origins of Hantaviruses Harbored by Bats, Insectivores, and Rodents
Source: PLoS Pathog. 2013 Feb 7;9(2):e1003159. doi: 10.1371/journal.ppat.1003159 (PMC3567184; doi:10.1371/journal.ppat.1003159)
Supplement: Table S6 — Specific primers used in this study. (DOC) [file ppat.1003159.s010.doc]

Table S6. Specific primers used in this study

| Type | Segment | Sequence(5’-3’) | Reference |
| --- | --- | --- | --- |
|  | Adaptor | TCAAGCTAGAGCTTACCG |  |
| LQUV/HUPV/YKSV/LHEV | L | ATG TAY GTI AGT GCW GAT GC (+) | 17 |
|  |  | TGC WGA TGC HAC IAA RTG GTC (+) | 35 |
|  |  | GRT TAA ACA TIC TYT TCC ACA THT C (-) | 17 |
|  |  | GCR TCR TCW GAR TGR TGD GCA A (-) | 35 |
| LQUV/ YKSV/LHEV | M | GGT CCG GGT GCA DSY TGT GAR GC (+) | 17 |
|  |  | GAA CCC CAD GCC CCI TCH AT (-) | 17 |
|  |  | TGT GTI CCW GGI TTY CAT GGI T (+) | 30 |
|  |  | CAT GAY ATC TCC AGG GTC HCC (-) | 30 |
| LQUV | S | TCW GGW GCH CCT GCA AAC AHC CA (-) | This study |
|  |  | GAG TTA ATG GTG WTT CWA TKC (-) | This study |
|  |  | AAC CAC AAR ARG CCG CCA AA (+) | This study |
|  |  | GAA ATA GRT TGC GTT TTA WGG (+) | This study |
|  | M | CAC ACA TCT CTG TTG CTA TTG AA (-) | This study |
|  |  | TGT TGC TAT TGA ATG GGC AG (-) | This study |
|  |  | TCC ATT AAA CGG TGG TGC CTT AC (+) | This study |
|  |  | GAG TAT TAG AAA CAA AAG ACT C (+) | This study |
| YKSV | S | TGC TTT AGA TAA GAA TAA GG (+) | This study |
|  |  | AGA ATA AGG ACT TTT TTA ACG (+) | This study |
|  |  | TGG CAA ATG GCG AAT CAA TTT (-) | This study |
|  |  | TTT GCA GGT ACA GAA TGA TG (-) | This study |
|  | M | CAC AAA GTT CAA CTG CTA TTG AA (-) | This study |
|  |  | AAC TGC TATT GAA TGA GCC A (-) | This study |
|  |  | CCC CAT GGA GGC TGG TGG GTT GA (+) | This study |
|  |  | AAG CTT ATG GCA ACA ATA GAC TC (+) | This study |
| LHEV | S | TAC CAC AAA AAG CAA TCG GG (+) | This study |
|  |  | GCA ATC GGG TGT TCT TTA TGG (+) | This study |
|  |  | CAG CAG ATG GGC TTT CTA TGT (-) | This study |
|  |  | ACT CAC AGG TGA ACA AGA GG (-) | This study |
|  | M | CAC ATA GCT CAA TCG CTA TTG AA (-) | This study |
|  |  | AAT CGC TAT TGA ATG AGC GA (-) | This study |
|  |  | ACC AATG GAA GGT GGT GGK TTA A (+) | This study |
|  |  | AAA CTG ATG GYT ACA MTT GAC TC (+) | This study |
| HUPV | S | GAC AGA CAA TTA ARG ATA AYA ARG G (+) | This study |
|  |  | CAG AAC TGC AGT ATG TGG CT (+) | This study |
|  |  | TGA GCT CAG GRT CCA TRT CAT CRC C (-) | This study |
|  |  | GAA AGC CAA TSA CWC CCA TTA CWG G (-) | This study |
|  |  | ATC TTT GCT GGT TCA CCT GAT (+) | This study |
|  |  | ACT GCT GAG GAG AAA CTG AAG (+) | This study |
